# Coumarin and Curcumin-Metal Complexes as Next-Generation Photosensitizers in Cancer Photodynamic Therapy

**DOI:** 10.3390/ijms27177585

**Published:** 2026-08-24

**Authors:** Siu Kan Law, Albert Wing Nang Leung, Chuanshan Xu

**Affiliations:** 1 Independent Researcher, Hong Kong SAR, China; 2 School of Graduate Studies, Lingnan University, Tuen Mun, Hong Kong SAR, China; albertleung@ln.edu.hk; 3 Guangzhou Municipal and Guangdong Provincial Key Laboratory of Molecular Target & Clinical Pharmacology, The NMPA and State Key Laboratory of Respiratory Disease, School of Pharmaceutical Sciences, Guangzhou Medical University, Guangzhou 511436, China

**Keywords:** natural ligand, coumarin, curcumin, metal complex, photodynamic therapy, cancer, immunogenic cell death, theranostic nanoplatforms

## Abstract

To explore the emerging role of natural ligands, specifically coumarin and curcumin, and their coordination with the transition metals ruthenium (Ru) and iridium (Ir) as photosensitizers (PSs) in photodynamic therapy (PDT) for cancer. This highlights the integration of natural compounds and transition metals to overcome limitations in photophysical properties, hypoxia tolerance, and clinical translation. Regarding PDT oncology, this examines an immunological effect on Ru/Ir complexes and natural ligand-metal hybrids. They induce immunogenic cell death (ICD) through reactive oxygen species (ROS) generation, calreticulin exposure, extracellular ATP release, and HMGB1 secretion. These damage-associated molecular patterns act as “danger signals” to recruit dendritic cells, prime CD8^+^ cytotoxic T-cells, and establish systemic antitumor immunity. This study compares natural ligand-metal complexes with conventional Ru(II)/Ir(III) complexes and clinical PSs to assess their translational potential as immune-activating agents in PDT oncology, as well as focusing on the integration of nanotechnology with natural ligand-metal complexes to enhance delivery, biocompatibility, and clinical translation. A narrative review was conducted of the literature published between 2010 and 2025 across multiple electronic databases, including WanFang Data, PubMed, ScienceDirect, Scopus, Web of Science, Springer Link, SciFinder, and CNKI, without language restrictions. Studies focusing on coumarin, curcumin, Ru(II), Ir(III), and PDT were analyzed. Extracted data included chemical structures, absorption and emission spectra, singlet oxygen yields, biological activities, and therapeutic outcomes. Comparative evaluation was performed between free natural ligands, their Ru(II)/Ir(III) complexes, and nanodelivery systems to assess efficacy, biocompatibility, and translational potential. Coumarin and curcumin exhibited intrinsic antioxidant, anti-inflammatory, and anticancer properties but were limited by short absorption/emission ranges, poor photostability, and low singlet oxygen yields, restricting preclinical application. Coordination with Ru(II) and Ir(III) significantly enhanced intersystem crossing, extended absorption into the near-infrared region, and improved singlet oxygen quantum yields (Φ_Δ_ up to ~0.78). These complexes demonstrated potent photocytotoxicity under normoxia and hypoxia, achieving IC_50_ values in the nanomolar range, which indicated organelle-specific targeting (mitochondria, lysosomes, ER), induced ICD, and synergized with checkpoint blockade. Nanocarrier encapsulation further improved solubility and tumor selectivity, and reduced systemic toxicity. Coumarin- and curcumin-based Ru/Ir complexes represent promising next-generation or immune activating PDT agents by combining natural pharmacological activity with superior photophysical performance. The ability to generate reactive oxygen species under hypoxia and achieve multimodal therapeutic effects positions them as strong candidates for clinical translation. Clinical approval of natural ligand-Ru/Ir complexes depends on rigorous safety, pharmacokinetic, and nanodelivery validation, but these complexes clearly extend PDT beyond local cytotoxicity toward durable immune protection. Future research should prioritize ligand engineering, nanotechnology integration, and translational models to bridge preclinical promise with safe and effective clinical applications.

## 1. Introduction

Metal-ligand complexes are recognized as multifunctional scaffolds for photoactivated therapeutic strategies that have notable applications in photodynamic therapy (PDT). These complexes consist of a central metal ion coordinated to organic or inorganic ligands, which modulate their photophysical and biological properties [1]. The metal ion typically belongs to transition metals such as ruthenium, iridium, copper, and zinc, or noble metals like platinum, gold, and silver [2]. Organic ligands are carbon-based molecules (e.g., porphyrins [3], BODIPY dyes [4]) and include natural ligand types such as coumarin and curcumin, whereas 5-aminolevulinic acid (5-ALA) [5] is a precursor that undergoes intracellular conversion to protoporphyrin IX (PpIX) for the active PS. In contrast, inorganic ligands are non-carbon species (e.g., nitrogen, sulfur, or chlorine) [6]. This distinction allows fine-tuning of light absorption, intersystem crossing, and reactive oxygen species (ROS) generation, thereby enhancing the therapeutic efficacy of PDT.

The advantages of employing metal-ligand complexes in PDT include: (a) Enhanced energy transfer, owing to heavy metal centers such as ruthenium (Ru) and iridium (Ir), which exhibit pronounced spin-orbit coupling due to their high atomic numbers. This ‘heavy atom effect’ significantly increases the efficiency of intersystem crossing (ISC), thereby facilitating the transition from the singlet excited state to the triplet state and promoting the generation of ROS [7]. (b) Improved tissue penetration, as organic PSs often require high-energy, invasive laser irradiation, whereas metal complexes can absorb in the near-infrared (NIR) region or beyond, enabling deeper penetration into biological tissues, including tumors [8]. (c) Oxygen independence for conventional PDT is limited in hypoxic tumors because it relies on molecular oxygen for ROS generation. Certain metal-ligand complexes address this constraint via oxygen-independent mechanisms. These complexes directly interact with and damage biomolecules upon light activation, such as cancer cell DNA, thereby inducing cytotoxicity even under low-oxygen conditions [9], and simultaneously triggering immunogenic cell death (ICD). The resulting release of calreticulin, ATP, and HMGB1 acts as an immune danger signal that recruits dendritic cells and primes CD8^+^ cytotoxic T cells, extending PDT efficacy beyond local cytotoxicity toward systemic antitumor immunity.

However, there are some drawbacks, including (a) potential toxicity, as Ru and Ir are heavy metals, which may cause off-target cytotoxicity or long-term tissue accumulation [10]; (b) excessive tissue penetration may activate photosensitizers outside the tumor margin, raising risks of off-target cytotoxicity and collateral damage [10]; (c) strong oxygen-independent activity requires a suitable delivery systems, otherwise they risk systemic toxicity through uncontrolled biomolecular interactions that damage healthy cells [11]. Thus, ligand design and delivery systems are emphasized to moderate oxygen-independent activity.

**Figure 1:**
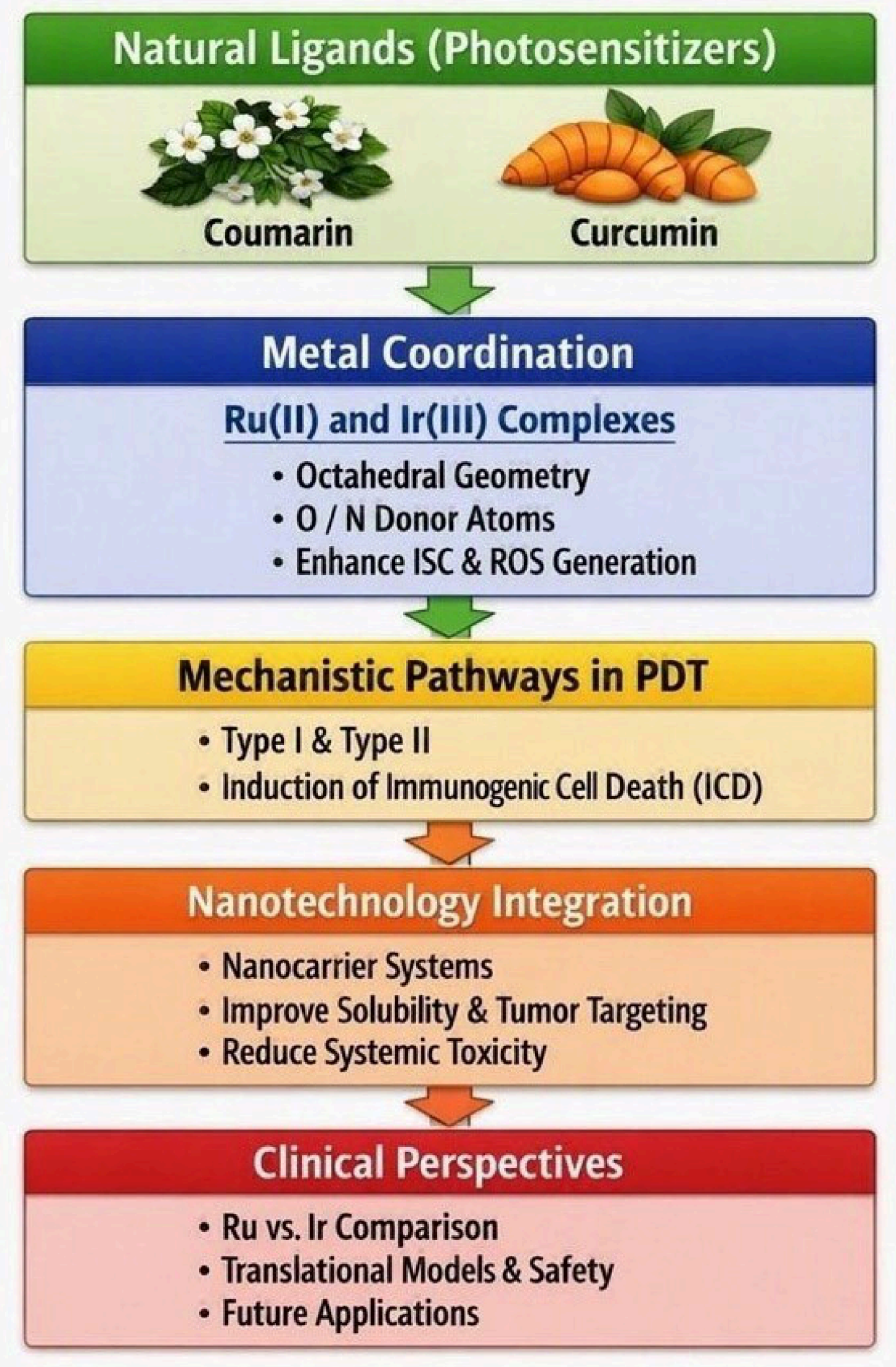
Overview of the article contents. The diagram introduces natural ligands (coumarin and curcumin) as photosensitizers, their coordination with Ru(II)/Ir(III) metals, mechanistic pathways in PDT, nanotechnology integration, and clinical perspectives through the logical flowchart.

A natural ligand is a possible choice in the metal complex for PDT, serving as a PS and a biocompatible scaffold to reduce systemic toxicity, improve solubility, and enhance bioavailability. Natural ligands often provide intrinsic bioactivity, such as anticancer, antioxidant, or antimicrobial effects, which synergize with the photophysical properties of a metal center to maintain therapeutic efficacy. The integration of these ligands into metal complexes enables precise modulation of absorption spectra, ISC efficiency, and ROS generation, while simultaneously buffering the adverse effects associated with heavy-metal toxicity. This review article aims to describe the role and properties of natural ligands, as well as the mechanisms of PDT against cancer, highlighting recent advances and identifying current challenges to provide ligand design and delivery strategies, which can enhance the safety and effectiveness of PDT in clinical applications (Figure 1). The Ru(II)/Ir(III) complexes coordinated with natural ligands such as coumarin and curcumin exhibit dual theranostic potential, combining PDT efficacy with bioimaging capabilities to generate ROS under hypoxia, trigger immunogenic cell death, and integrate with nanodelivery systems. Advanced preclinical models are critical to bridge the gap between laboratory innovation and safe, effective cancer therapy.

Representative examples include coumarin and curcumin, natural ligands derived from traditional Chinese medicine. They provide intrinsic antioxidant, anti-inflammatory, and anticancer activities, but their limited photophysical properties restrict direct PDT application. Coordination with Ru(II) or Ir(III) metals significantly improves absorption spectra, singlet oxygen yields (Φ_Δ_), and hypoxia tolerance, as well as nanocarrier encapsulation enhances biocompatibility for tumor selectivity. These advances highlight the translational potential of natural ligand-metal complexes as dual therapeutic and diagnostic agents in cancer PDT. This review primarily focuses on coumarin and curcumin as representative natural ligands for PDT, while other natural ligands are briefly mentioned to provide context.

## 2. Search Strategy

This narrative review was conducted using search strings across major databases, including PubMed, Scopus, Web of Science, and the China National Knowledge Infrastructure (CNKI). The search covered publications from 2010 to March 2025, guided by keywords such as “Ruthenium” OR “Iridium” OR “Coumarin” AND “Curcumin” OR “Photodynamic Therapy” OR “Cancer.” The study findings were presented and summarized without language restrictions (Supplementary File S1). Regarding the search strategy, it specifically targeted studies on coumarin- and curcumin-derived ruthenium and iridium complexes for cancer photodynamic therapy.

Records were screened in two stages: (1) titles and abstracts were reviewed to exclude irrelevant studies, and (2) full texts were assessed for eligibility. Inclusion criteria comprised studies that investigated coumarin- and curcumin-derived Ru(II) and Ir(III) complexes for PDT in oncology. Exclusion criteria included conference abstracts without full papers, non-oncology applications, and studies lacking methodological detail.

The selection workflow identified 312 records: 102 from PubMed, 78 from Scopus, 92 from Web of Science, and 40 from CNKI. After removing 56 duplicates, 256 records remained for screening. Title and abstract screening excluded 198 records as irrelevant. The remaining 58 full texts were assessed for eligibility, of which 23 were excluded (non-oncology focus, insufficient methodological detail, or conference abstracts only). Finally, 35 studies were selected to fulfill the inclusion criteria and were synthesized in this narrative review (Supplementary File S1, Table S1).

This study is a narrative review as it synthesizes and interprets existing evidence on coumarin- and curcumin-derived ruthenium and iridium complexes in PDT without conducting a quantitative meta-analysis. It integrates findings from diverse sources, highlighting photophysical properties, hypoxia tolerance, nanodelivery strategies, and immunological effects such as ICD. Unlike systematic reviews, which follow rigid protocols and statistical pooling, the narrative approach allows flexibility to compare natural ligands, Ru/Ir complexes, and clinical photosensitizers, while emphasizing mechanistic insights and translational potential. The methodology involved structured database searches, screening, and thematic categorization, but the focus remained on conceptual integration rather than exhaustive quantitative synthesis. This format is particularly suited to emerging fields, where heterogeneous study designs and preclinical data require contextual interpretation to identify trends, challenges, and future directions for ligand engineering and clinical translation in PDT oncology.

## 3. Natural Ligands

Natural ligands are usually biologically derived and carbon-based molecules that can coordinate with central metal ions to form stable complexes. Many of these ligands originate from traditional Chinese medicinal plants, leading to biocompatible PSs in PDT. Unlike synthetic ligands, natural ligands often possess intrinsic pharmacological activities that complement traditional Chinese medicine (TCM) theory, such as antioxidant, anti-inflammatory, and anticancer effects, which synergize with the photophysical properties of the metal center (Table 1). By conjugating natural ligands with metal ions, as a PS, the resulting complexes may reduce severe side effects associated with heavy-metal toxicity, while simultaneously enhancing the selectivity and therapeutic efficacy of PDT for cancer [12].

## 4. Chemical Structure and Photophysical Features

Natural ligands have unique chemical structures that exhibit photophysical features to directly determine their efficiency as PSs, including absorption (λ_abs_), excitation (λ_ex_), emission (λ_em_), and singlet oxygen quantum yield (Φ_Δ_). These properties are influenced by the ligand’s structure and the coordinating metal center [8].

Coumarin contains a benzopyrone, which shows blue fluorescence but poor singlet oxygen yield. Curcumin is a polyphenolic β-diketone that exhibits moderate ROS generation with broad emission (Table 2).

## 5. Metal Complexes

Ruthenium (Ru) and iridium (Ir) are rare, heavy transition metals that are usually combined with organic molecules to form metal complexes because of their unique chemical behavior. These electronic configurations and relativistic effects lead to the formation of stable coordination complexes. The d-orbitals enable versatile bonding with organic ligands, allowing photophysical properties such as absorption wavelength, redox potential, and excited-state lifetime [25]. The transition metal Ru or Ir complex has the potential to (a) enhance spin-orbit coupling, which facilitates the ISC for the non-radiative transition from the singlet excited state (S_1_) to the triplet excited state (T_1_) [26]; (b) stabilize the reactive intermediate to improve ROS yield and photostability [27]; (c) extend the absorption and emission into the NIR region for deeper tissue penetration [28].

Ru(II) and Ir(III) complexes typically adopt octahedral or pseudo-octahedral coordination geometries, which have coordination numbers ranging from 6 to 8 depending on the ligand environment. Ru(II) polypyridyl complexes exhibit stable 6-coordinate octahedral structures, where nitrogen donor atoms from bipyridine or phenanthroline ligands dominate. Ir(III) cyclometalated complexes favor octahedral geometries, stabilized by strong C^N ligands. Natural ligands such as coumarin or curcumin are inserted, and their oxygen donor groups (carbonyl, hydroxyl, β-diketone moieties) coordinate to the metal center, forming mixed-ligand complexes. This coordination not only stabilizes the complex but also modulates absorption spectra, ISC efficiency, and Φ_Δ_ yields. Thus, the geometry and donor atom type are critical determinants of photophysical behavior and PDT efficacy.

### 5.1. Mechanistic Pathways of Metal Complexes in PDT

**Figure 2:**
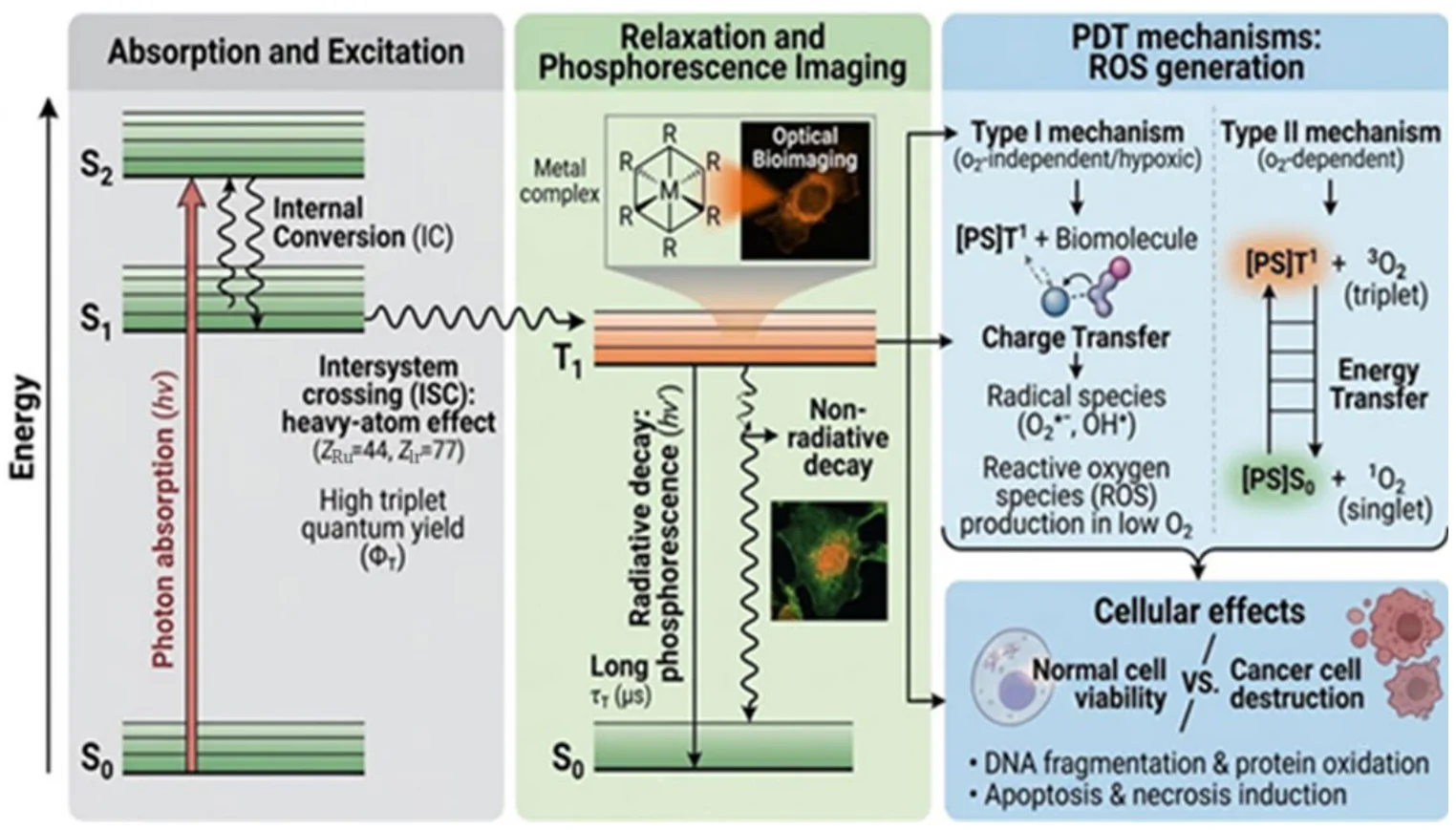
Photophysical and biological mechanisms of metal complexes for PDT.

Generally, the Ru(II) or Ir(III) complex acts as a dual theranostic agent in PDT and bioimaging, which is divided into three stages (Figure 2):

#### 5.1.1. Absorption and Excitation

Photon absorption (hv) excites the PS from the ground singlet state (S_0_) to the excited singlet state (S_2_). Subsequent internal conversion (IC) relaxes the system to (S_1_). The prominent heavy-atom effect of the transition metal centers, such as Z_Ru_ = 44 and Z_Ir_ = 77, drives strong spin-orbit coupling. This enhances ISC, leading to an exceptionally high triplet quantum yield (Φ_Δ_) into the triplet state (T_1_) [29].

#### 5.1.2. Relaxation and Phosphorescence Imaging

Upon reaching the T_1_ state, the metal complex undergoes competitive relaxation. Radiative decay back to S_0_ produces long-lived phosphorescence lifetimes τT in microseconds. This delayed emission bypasses short-lived cellular autofluorescence, making these complexes highly effective for targeted optical bioimaging [30]. The state deactivates via non-radiative decay pathways alternatively.

#### 5.1.3. ROS Generation from PDT

The excited triplet state (T_1_) interacts with the cellular microenvironment to generate ROS via two mechanisms [29]: Type I mechanism (O_2_-independent) involves charge transfer between the PS (T_1_) and neighboring biomolecules, producing destructive radical species (O_2_^•−^, •OH). This pathway remains highly active in hypoxic (low-oxygen) tumor domains. Type II mechanism (O_2_-dependent) relies on direct energy transfer from PS (T_1_) to ground-state triplet oxygen (^3^O_2_), generating highly cytotoxic singlet oxygen (^1^O_2_). The resulting intracellular burst of ROS leads to cancer cell destruction while sparing normal cell tissue. Downstream cytotoxic effects include direct DNA fragmentation, severe protein oxidation, and the rapid execution of apoptosis and necrosis [31]. Therefore, Ru and Ir metal-based complexes have attracted considerable attention in recent years for their potential in PDT to explore anticancer activity.

Ru(II) complexes exhibited broad visible absorption, red phosphorescence, and efficient ^1^O_2_ generation, which possessed potent photocytotoxicity with IC_50_ values in the low micromolar to nanomolar range under irradiation, effective even in hypoxia [32,33,34,35,36,37,38,39,40,41,42]. Mechanisms include apoptosis, ferroptosis, immunogenic cell death, and synergy with chemotherapy. The strategy involved designations such as Ru-Pt conjugates, binuclear Ru-Re complexes, and two-photon excitation systems to enhance tumor selectivity and overcome drug resistance. For example, novel Ru(II) complexes triggered CD8^+^ T cell antitumor responses with inhibition rates up to 97.3% [32], and polypyridyl complexes achieved a high ΦΔ of ~0.67 and strong cytoplasmic localization [40].

Ir(III) complexes demonstrated strong red/NIR luminescence, a high Φ_Δ_ of ~0.78, and organelle-specific targeting of the mitochondria [43], lysosomes, and endoplasmic reticulum to achieve remarkable photocytotoxicity under normoxia and hypoxia. The IC_50_ values were as low as 0.012 µM [44]. The strategy involved designations such as nanoprodrug encapsulation [45], protein modification [46], NIR activation [47,48], and ferroptosis induction [49,50]. These complexes were given a versatile, multimodal, and hypoxia-tolerant PDT platform for diverse cancers [51,52,53].

## 6. Natural Ligands for PDT

Natural ligands serve as multifunctional, biocompatible scaffolds bridging traditional medicine and modern molecular oncology [1]. These biologically derived carbon-based structures frequently originate from traditional Chinese medicine sources [12], including *Peucedanum decursivum* (Qian Hu) and *Curcuma longa* L., which offer intrinsic antioxidant, anti-inflammatory, and anticancer activities [13,18], as well as properties that operate in direct synergy with central metal centers [12].

Coumarin and curcumin are natural ligands coordinated with transition or noble metals, optimizing the pharmacological profile [2]. Specifically, they act as molecular buffers to lessen severe off-target heavy-metal toxicity [12], and their structural diversity enables precise fine-tuning of photophysical characteristics [8]. They extend absorption spectra toward the NIR region for deeper tissue penetration [28], which elevates ISC efficiency and maximizes ROS quantum yields [26]. Thus, natural ligands address critical translational challenges as they improve aqueous solubility, enhance intracellular bioavailability, and establish a multi-targeted therapeutic platform, as well as overcome hypoxia-induced resistance while safeguarding normal cells [44].

Coumarin-based PDT exemplified this versatility. COUPY coumarins exhibited pronounced mitochondria-targeting properties, achieving nanomolar IC_50_ values under visible-light irradiation, generating ROS to induce apoptosis and autophagy [54]. Nanoencapsulation of coumarin PS in polyurethane-polyurea significantly reduced dark toxicity and enhanced photoactivity of the delivery systems to optimize therapeutic efficacy [55]. A dual-action coumarin-camptothecin polymer combined drug release with ROS generation, achieving strong cancer cell killing upon 365 nm excitation [56]. Similarly, Calixarene-based nanostructures encapsulating Coumarin 6 facilitated tumor-cell imaging and induced photo-cytotoxicity, accompanied by efficient ^1^O_2_ generation [57]. A cinnamoyl-coumarin-RGD conjugate targeted MCF-7 tumor destruction, with a 0.62 singlet oxygen yield [58]. The TiO_2_-coumarin nanoconjugate enables effective combination therapy through broad visible light activation in breast cancer [59]. Additionally, carbazole-coumarin conjugates offered mitochondrial targeting in leukemia cells, reducing light cytotoxicity to 0.1 µM (Table 3) [60]. These studies indicated the adaptability of coumarin scaffolds in PDT, where structural modifications and nanocarrier strategies enhance selectivity, reduce side effects, and improve anticancer outcomes.

Curcumin-based PDT demonstrated broad anticancer efficacy across melanoma, breast, head and neck, cervical, nasopharyngeal, gastric, and ovarian cancers. Studies reveal consistent ROS generation, apoptosis induction, and IC_50_ reduction under light irradiation. Nanotechnology formulations, including nanoemulsions, EGFR-targeted chitosan/TPP nanoparticles, and PLGA nanoparticles, improved curcumin’s solubility, stability, and tumor selectivity. These systems provided site-specific activation, enhanced caspase-3/7 activity, reversed multidrug resistance, and impaired oxidative metabolism. Curcumin’s absorption from 380 to 500 nm with λ_max_ ~440 nm and green-yellow emission from ~500 to 550 nm support effective PDT under blue light. Dark cytotoxicity was observed, but photocytotoxicity was significantly stronger, and IC_50_ values were reduced to ~7–12 µM (Table 4) [61,62,63,64,65,66,67,68]. Curcumin’s dual function as a chemotherapeutic agent and PS, when combined with nanoparticle delivery systems, establishes it as a versatile candidate for clinical PDT development, enhancing therapeutic efficacy and minimizing adverse effects through targeted formulations.

## 7. Natural Ligands with Ru(II)/Ir(III) Complexes for PDT

The combination of Ru(II) and Ir(III) complexes with a natural ligand is a powerful strategy to enhance the effectiveness of PDT, because they are heavy transition metals that provide strong spin-orbit coupling and long-lived triplet states, which facilitate ISC and efficient ROS generation. Natural ligands, including coumarin, curcumin, and pheophorbide a, contribute intrinsic bioactivity, biocompatibility, and improved pharmacokinetics. This synergy enables the design of multifunctional PSs to merge the photophysical robustness of metal centers with the therapeutic selectivity of plant-derived scaffolds.

Ru(II) and Ir(III) coumarin or curcumin complexes demonstrated potent PDT activity with broad visible absorption and efficient ROS generation. Ru(II)-coumarin conjugates achieved micromolar IC_50_ values under normoxia and hypoxia, which possessed a Φ_Δ_ of ~0.40–0.50, highlighting hypoxia tolerance [69]. Ru-COUBPY complexes exhibited exceptional nanomolar potency for IC_50_ ≈ 0.0074 µM under 645 nm irradiation [70]. Coumarin-modified Ru complexes induced signaling downregulation with a moderate Φ_Δ_ of ~0.2–0.3 [71], and Ru-polyimine-coumarin systems exhibited strong DNA photocleavage and a Φ_Δ_ of 0.67 [72]. Ir(III) coumarin conjugates triggered apoptosis/autophagy with a Φ_Δ of_ ~0.6–0.7 [73], and Ir-COUPY complexes reached a Φ_Δ_ of ~0.81 with submicromolar IC_50_ values [74]. Highly efficient Ir(III) coumarin redox catalysts displayed ultralow IC_50_ values from 0.01 to 0.03 µM [75], Ir-COUPY ferroptosis inducers combined Type I/II ROS generation [76], and Coumarin 6-based Ir(III) photocatalysts demonstrated photo-toxicities, which localized in the mitochondria, generated ROS, and oxidized NADH (Table 5) [77]. Curcumin-based Ru/Ir complexes enhanced ROS production, apoptosis, and dual PDT/PAC mechanisms, with a Φ_Δ_ of up to 0.98 (Table 6) [78,79,80]. These findings underscore the versatility of Ru/Ir coumarin and curcumin complexes as hypoxia-tolerant, multimodal PDT agents.

### 7.1. Photophysics and PDT Mechanisms

#### 7.1.1. Coumarin and Coumarin Complex

Coumarin undergoes light absorption to reach the excited singlet state (S_1_), resulting in competitive decay via fluorescence (S_1_ → S_0_) or ISC to the triplet state (T_1_). Coordinating coumarin with Ru(II) or Ir(III) mediates a prominent Metal-to-Ligand Charge Transfer (MLCT) state, which drastically enhances the ISC rate from S_1_ to T_1_ via the heavy-atom effect. The triplet state subsequently releases energy via the Type II pathway to generate singlet oxygen (^1^O_2_) or undergoes electron transfer via the Type I pathway to produce free radicals [81].

#### 7.1.2. Curcumin and Curcumin Complex

Curcumin transits the absorption energy into a Charge Transfer (CT) state to undergo rapid, non-radiative decay from S_1_ to CT to S_0_, which suppresses fluorescence and ISC efficiency. Complexation with Ru(II)/Ir(III) alters the pathway to override non-radiative decay through the MLCT state. This boosts ISC efficiency to the T_1_ state, allowing the complex to efficiently activate Type I and Type II pathways to generate high yields of ROS [82].

#### 7.1.3. Coumarin and Curcumin

Coumarin undergoes direct radiative deactivation (fluorescence) or ISC, but curcumin predominantly relaxes via a low-lying CT state with different excited-state relaxation [83,84]. Meanwhile, coumarin readily retains energy for emission or triplet conversion, but curcumin undergoes rapid, non-radiative thermal decay (S_1_ to CT to S_0_), which heavily suppresses its native fluorescence and triplet state excitation [85]. The energy loss mechanism is distinct.

#### 7.1.4. Coumarin Complex and Curcumin Complex

**Figure 3:**
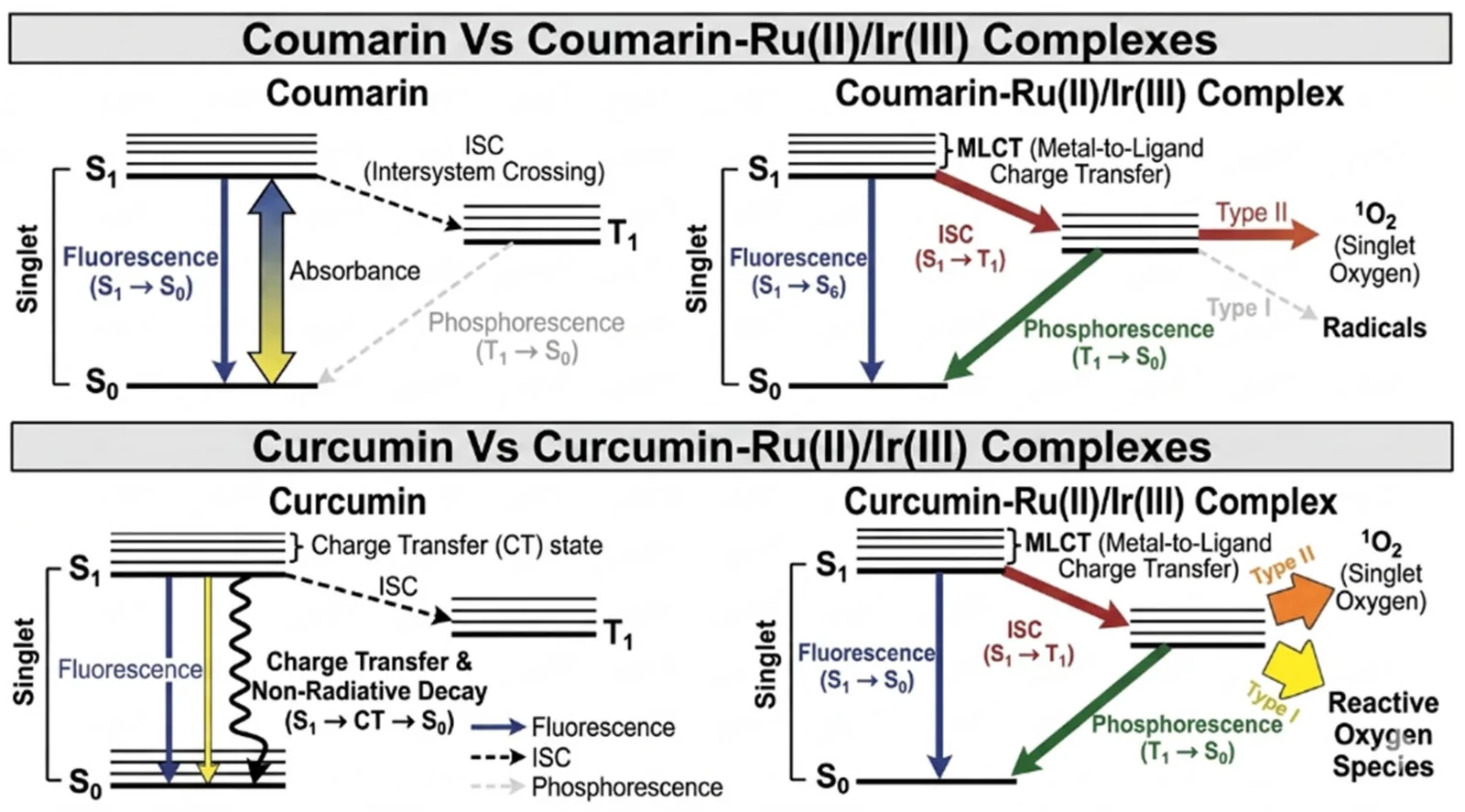
Photophysics and PDT mechanisms of coumarin and curcumin ligands versus Ru(II)/Ir(III) metal complexes.

The coordination of coumarin to Ru(II) or Ir(III) centers induces a prominent MLCT state, thereby accelerating the ISC rate [70]. Conversely, the MLCT manifold in curcumin complexes suppresses the ligand’s intrinsic non-radiative decay, redirecting the relaxation pathways of the excited state. This divergence dictates the phototherapeutic mechanisms, including coumarin complexes, which distinctly partition triplet energy into Type II singlet oxygen (^1^O_2_) generation and Type I radical pathways [86], whereas curcumin complexes leverage facilitated IS to generate a high flux of ROS via a synergistic Type I/II modality [87]. Thus, transition metal complexation serves as a strategy to re-engineer ligand photophysics, converting poorly active scaffolds into potent PSs for PDT (Figure 3).

### 7.2. Duality of Natural Bioactivity and Metal-Driven Photophysics

The emergence of natural ligand-metal complexes introduces a paradigm shift in PS design for PDT. Traditional organic PSs are effective but often suffer from limited absorption ranges, poor photostability, and low Φ_Δ_. Natural ligands such as coumarin and curcumin provide intrinsic pharmacological activities, including antioxidant, anti-inflammatory, and anticancer effects that synergize with PDT mechanisms [12,23,24]. However, the direct application is restricted by modest photophysical performance. Coordination with transition metals such as Ru(II) and Ir(III) fundamentally alters this issue. The heavy-atom effect enhances ISC, extending absorption into the NIR region and significantly improving Φ_Δ_ [7,8,29]. This duality of natural bioactivity combined with metal-driven photophysical optimization creates next-generation PSs capable of potent photocytotoxicity under both normoxic and hypoxic conditions. These complexes not only destroy tumor cells but also induce immunogenic cell death, releasing calreticulin, ATP, and HMGB1 to activate systemic antitumor immunity. When integrated with nanocarrier delivery systems, they achieve improved solubility, tumor selectivity, and reduced systemic toxicity. Collectively, the innovative design strategy bridges traditional bioactivity with advanced photophysics, positioning natural ligand-metal complexes as immune-activating PDT agents with strong translational potential.

## 8. Immune-Activating Agents/Photosensitizers

Ru or Ir metal complexes are advancing oncology through PDT measured by immune modulation (Table 7).

### 8.1. Ruthenium(II) Complexes

Ru(II) complexes generate ^1^O_2_ and ROS, leading to oxidative stress and endoplasmic reticulum damage. This cascade promotes the release of damage-associated molecular patterns (DAMPs), including calreticulin exposure, ATP secretion, and HMGB1 release. These signals act as “danger cues” to recruit and activate dendritic cells, which process tumor antigens and present the corresponding signal via MHC-I, thereby priming CD8^+^ cytotoxic T-cells. Ru-based PDT not only caused direct apoptosis and necrosis of HeLa cells but also initiated systemic immune responses, highlighting its dual role in tumor destruction and immune activation. More importantly, Ru(II) complexes showed efficient cellular uptake and strong phototoxicity under visible light, and remained relatively inert in the dark, underscoring their therapeutic selectivity. This study provided compelling evidence that Ru complexes can serve as next-generation PDT agents capable of combining localized cytotoxicity with durable antitumor immunity and as promising candidates for clinical translation in oncology [88].

Recently, Ruthenium(II) polypyridyl complexes have been investigated as novel PSs for colorectal cancer PDT. These complexes efficiently generate singlet oxygen ^1^O_2_ and ROS, leading to oxidative stress and tumor cell death upon red-light irradiation. The study demonstrated that Ru(II) complexes trigger immunogenic cell death (ICD), characterized by calreticulin exposure, ATP release, and HMGB1 secretion. These damage-associated molecular patterns recruit dendritic cells, which process tumor antigens and activate CD8^+^ cytotoxic T-cells, thereby establishing systemic antitumor immunity. In vivo models confirmed that Ru-based PDT not only suppressed primary colorectal tumors but also enhanced immune checkpoint blockade therapy, converting “cold” tumors into “hot” ones responsive to immunotherapy. This dual mechanism has direct phototoxicity and immune activation, which positions Ru(II) complexes as promising agents for photodynamic immunotherapy, offering localized tumor destruction and durable immune protection in colorectal cancer [89].

### 8.2. Iridium(III) Complexes

Iridium(III) polypyridyl complexes are advanced PDT agents with unique photophysical properties. Their long-lived triplet excited states allow efficient energy transfer to molecular oxygen, generating ^1^O_2_ and ROS that damage lipids, proteins, and nucleic acids. This oxidative stress ICD is marked by calreticulin exposure, extracellular ATP release, and HMGB1 secretion. These signals recruit and activate dendritic cells, which present tumor antigens to CD8^+^ T-cells, stimulating cytotoxic immune responses. In vivo studies have indicated that Ir(III) complexes not only suppress tumor growth through direct PDT cytotoxicity but also enhance systemic antitumor immunity by increasing CD8^+^ infiltration into tumors. Furthermore, Ir complex-based PDT has been reported to synergize with immune checkpoint blockade therapies, effectively converting non-responsive tumors into immunologically active ones. This dual mechanism underscores the potential of Ir complexes to achieve immediate tumor destruction and durable immune protection, which is highly attractive for clinical translation in oncology [90].

Another study presents novel 1-methyl-4-phenylpyridinium-functionalized Ir(III) complexes for cancer PDT. The complex localizes to mitochondria, where light activation disrupts the respiratory chain, leading to excessive ROS generation. This oxidative stress triggers ferritinophagy, a selective autophagic process that degrades ferritin, releasing free iron and amplifying ROS production. The cascade culminates in ICD, marked by calreticulin exposure, ATP release, and HMGB1 secretion. These damage-associated molecular patterns recruit dendritic cells and activate CD8^+^ cytotoxic T-cells, establishing systemic antitumor immunity. In vivo models confirmed potent PDT efficacy, with significant tumor regression and enhanced immune infiltration. The Ir(III) PS remained relatively non-toxic in the dark, underscoring its therapeutic selectivity [91]. This study highlights iridium complexes as promising PDT agents to combine mitochondrial targeting, ferroptosis-like mechanisms, and durable immune activation for oncology applications.

**Figure 4:**
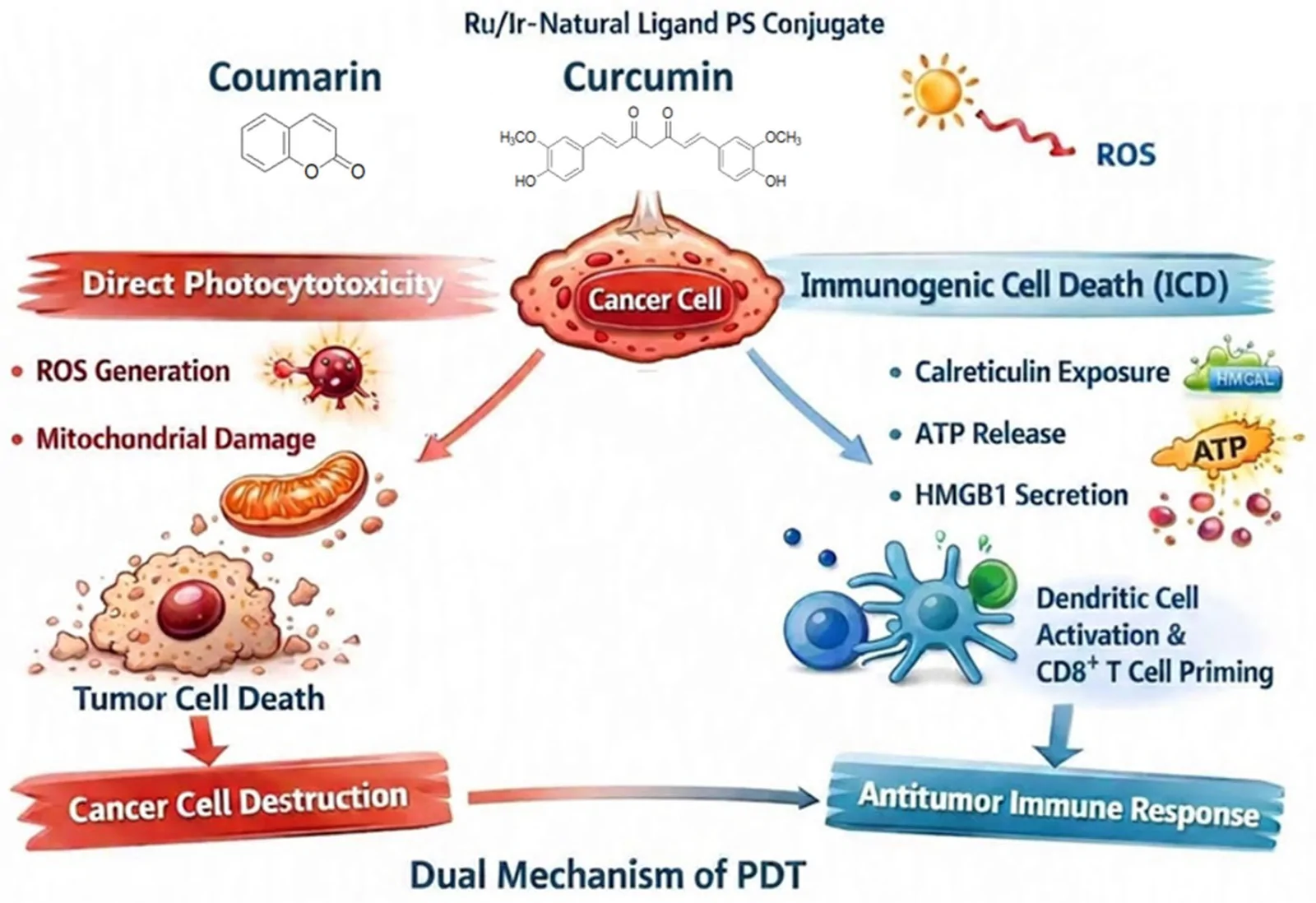
Dual mechanism of Ru/Ir-natural ligand PSs in PDT.

The dual mechanism of Ru/Ir-natural ligand conjugates integrates direct photocytotoxicity and ICD, as illustrated in Figure 4. These conjugates bridge photochemical cytotoxicity and immune modulation, achieving immediate tumor eradication and long-term immune protection.

## 9. Nanotechnology Integration and Mechanisms in PDT

Nanotechnology has become indispensable for advancing PDT, particularly for natural ligand-metal complexes such as coumarin- and curcumin-derived Ru/Ir systems. These complexes exhibit potent photocytotoxicity and ICD induction, but their clinical application is limited by poor solubility, rapid clearance, and off-target toxicity. Nanocarrier encapsulation directly addresses these problems by improving PK, enhancing tumor selectivity, and reducing systemic side effects. Polymeric nanoparticles, liposomes, dendrimers, and cubosomes have been widely employed to stabilize Ru/Ir complexes, prolong circulation time, and provide protection from premature degradation. Nanocarriers exploit the enhanced permeability and retention (EPR) effect for passive tumor targeting, and active targeting can be achieved through ligand modification, such as folate or antibody conjugation [41,45]. These strategies ensure preferential accumulation in tumor tissues to spare normal cells, thereby improving therapeutic indices. Nanotechnology enables co-delivery systems, where Ru/Ir complexes are combined with chemotherapeutic agents or immune modulators to achieve synergistic effects. For example, Ru-Pt conjugates encapsulated in nanocarriers demonstrated nanomolar cytotoxicity against drug-resistant cancers [36]. Stimuli-responsive nanoplatforms, including pH-sensitive, redox-responsive, and hypoxia-responsive carriers, further refine selectivity by releasing payloads specifically within the tumor microenvironment [47]. Nanocarriers aim to enhance theranostic potential, as Ru/Ir complexes exhibit long-lived phosphorescence lifetimes suitable for optical tracking, enabling real-time imaging to guide PDT [30,40].

Comparing nanotechnology applied to natural ligand-metal complexes with other PDT strategies, some important mechanistic distinctions emerge. In Ru/Ir-natural ligand systems, nanotechnology primarily enhances dual functionality, which improves photophysical performance through metal coordination while preserving the intrinsic pharmacological activity of the natural ligands. This dual mechanism amplifies the Type I, oxygen-independent radical generation, and Type II, singlet oxygen production, pathways, while simultaneously boosting ICD signals such as calreticulin exposure and ATP release [32,38].

**Figure 5:**
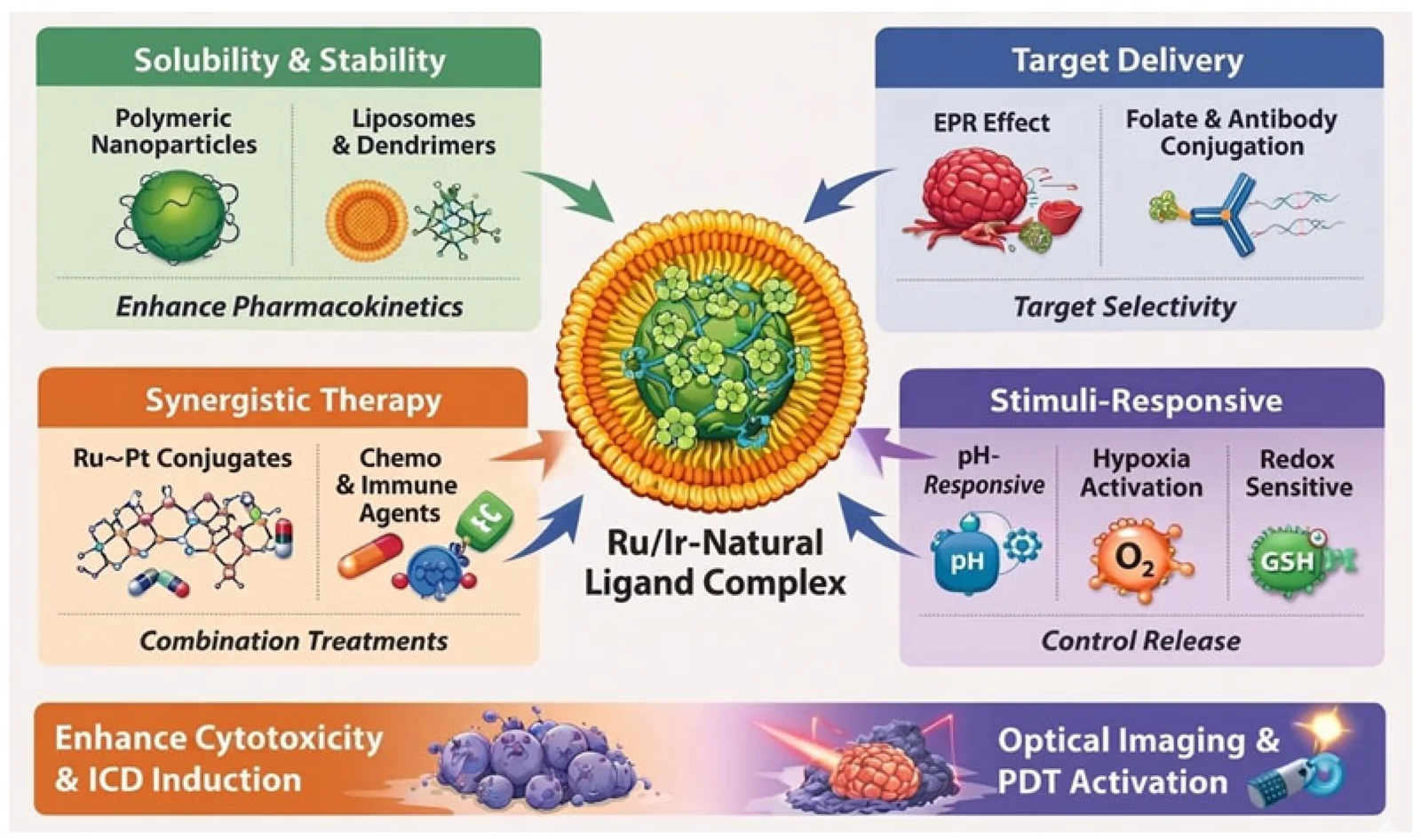
Nanotechnology of Ru/Ir-natural ligand complex in PDT.

Recent studies confirm that nanotechnology integration enhances photophysical performance and immune activation. Cyclometalated Ir(III) complexes in nanocarriers demonstrated robust type I photoreactivity under hypoxia [92], and Ru(II)/Ir(III) hybrids with β-carboline ligands indicated potent anticancer PDT activity and organelle targeting [93]. Biomimetic nanoparticles and exosome-based delivery systems emphasize their translational promise when combined with natural product PSs, highlighting improved tumor selectivity and systemic immunity [94,95]. Similarly, multifunctional nanoplatforms responsive to tumor microenvironment cues (e.g., glutathione depletion, hypoxia relief) have demonstrated cascade-amplified PDT efficacy and immune synergy [96,97] (Figure 5).

Nanotechnology transforms natural ligand-metal complexes into clinically viable PDT agents to improve solubility, tumor selectivity, and immune synergy. Nanocarrier systems bridge the gap between preclinical promise and translational oncology. Compared with conventional nanoplatforms that emphasize luminescence and penetration, Ru/Ir-natural ligand complexes uniquely unite optical precision, pharmacological bioactivity, and immune activation. Future research should prioritize multifunctional nanoplatforms to combine PDT efficacy with immune checkpoint blockade, hypoxia relief, and imaging guidance, positioning Ru/Ir-natural ligand complexes as next-generation immune-activating PDT agents.

## 10. Nanocarrier-Based Delivery Systems

Nanocarrier-based delivery systems have emerged as a critical innovation to enhance the therapeutic index of Ru/Ir-natural ligand complexes in PDT. These complexes exhibit potent photocytotoxicity and ICD, but clinical translation is hindered by poor solubility, nonspecific biodistribution, and risks of systemic toxicity [9,11]. Encapsulation with nanocarriers such as liposomes, polymeric nanoparticles, dendrimers, and nanoemulsions provides a versatile strategy to improve pharmacokinetics, tumor selectivity, and safety. Liposomal formulations stabilize hydrophobic ligands and enable passive accumulation via the EPR effect, and polymeric nanoparticles offer controlled release and surface modification for active targeting [32,40].

**Figure 6:**
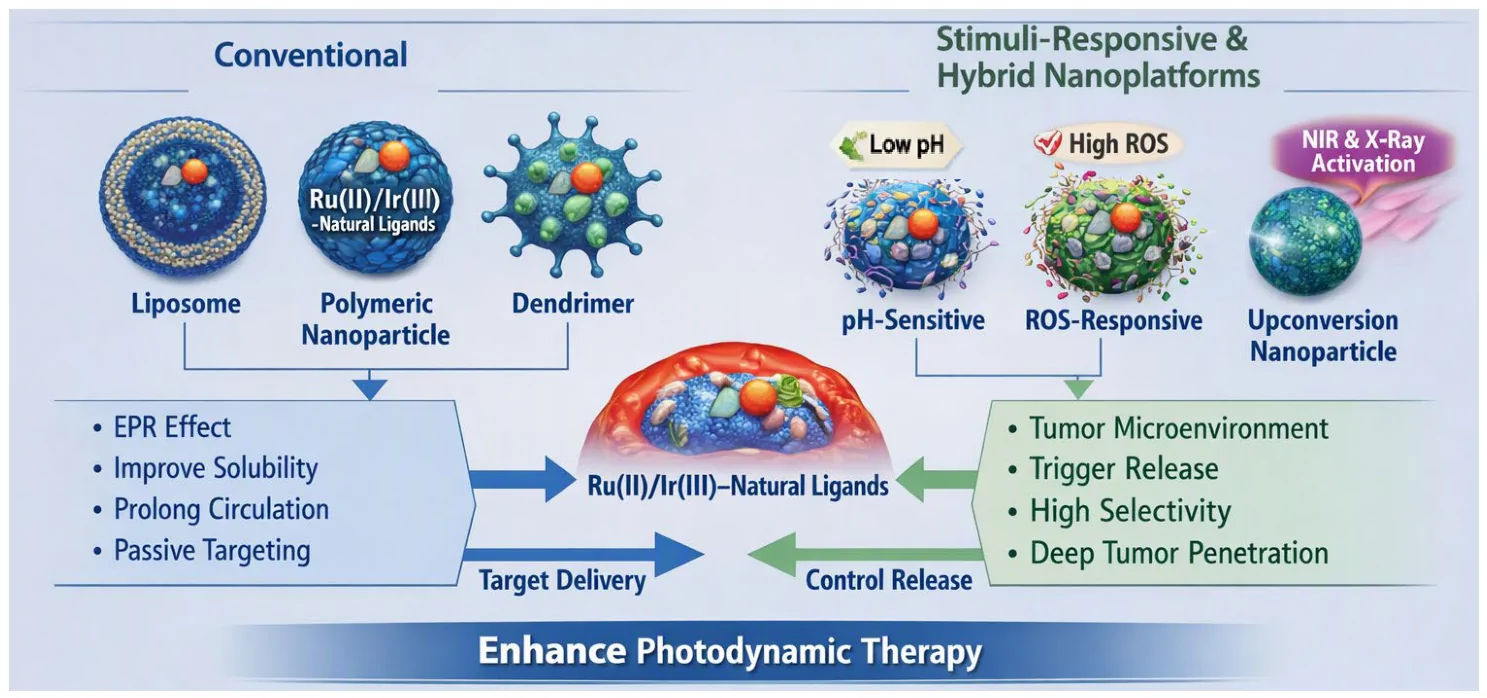
Delivery systems in PDT.

Beyond conventional carriers, stimuli-responsive nanoplatforms represent the next frontier in PDT delivery. pH-sensitive systems exploit the acidic tumor microenvironment to trigger selective release, ROS-responsive carriers amplify therapeutic efficacy by releasing payloads in response to oxidative bursts generated during PDT, and enzyme-triggered nanocarriers degrade in the presence of tumor-associated proteases to achieve site-specific activation. Hybrid nanoplatforms, such as upconversion nanoparticles or X-ray scintillator-based systems, extend activation wavelengths into the NIR or ionizing radiation domains, thereby overcoming tissue penetration limits and enabling treatment of deep-seated tumors (Table 8) (Figure 6) [45].

These advanced nanodelivery strategies not only improve tumor accumulation and minimize off-target effects but also integrate diagnostic and therapeutic functions, positioning Ru/Ir-natural ligand complexes as true theranostic agents. By combining natural bioactivity, metal-driven photophysics, and intelligent nanocarrier engineering, PDT evolves into a multimodal, immune-activating platform with strong translational potential. Nanocarrier-based delivery systems are a cornerstone for bridging preclinical promise with safe and effective clinical application, but further investigation is required.

## 11. Clinical Photosensitizers

Clinically approved PSs such as photofrin, verteporfin, and talaporfin have established PDT as a viable cancer therapy (Table 9).

Photofrin^®^ (Porfimer sodium) was the first globally approved PDT PS by the Food and Drug Administration for the treatment of esophageal cancer, non-small cell lung cancer (NSCLC), and high-grade dysplasia in Barrett’s esophagus [98]. This is the first-generation PS with a shorter wavelength, prolonged photosensitivity, and less selectivity. Its mechanism is based on selective accumulation in tumor tissues, followed by activation with red light at ~630 nm. The transitions from the ground singlet state to an excited triplet state transfer energy to molecular oxygen, producing ^1^O_2_ and other ROS upon irradiation. These ROS oxidize cellular macromolecules such as lipids, proteins, and nucleic acids, leading to structural damage and cell death [99]. The cytotoxic effects include apoptosis through mitochondrial disruption and necrosis via membrane damage [100]. Photofrin-PDT causes vascular shutdown within tumors, reducing oxygen and nutrient supply, which further enhances tumor destruction [101]. The disadvantages of using photofrin include a short absorption wavelength that restricts tissue penetration to about 5–10 mm, and patients often experience prolonged cutaneous photosensitivity [98]. However, these agents have prolonged skin photosensitivity, shallow tissue penetration, and dependence on oxygen for ROS generation, which reduces efficacy in hypoxic tumors.

Verteporfin (Visudyne^®^) is a benzoporphyrin derivative PS developed as a second-generation PS for PDT. Administered intravenously in a liposomal formulation, it accumulates in tumor tissues via low-density lipoprotein receptor uptake, improving selectivity. Verteporfin absorbs photons and undergoes intersystem crossing to a triplet state upon illumination with red light at ~689 nm. This excited state transfers energy to molecular oxygen, producing ^1^O_2_ as the predominant cytotoxic species. The generated ROS oxidize lipids, proteins, and nucleic acids, leading to apoptosis and necrosis of tumor cells [102]. Verteporfin-PDT damages tumor vasculature, causing vessel occlusion and restricting nutrient supply, which enhances tumor destruction [103]. Compared to photofrin, verteporfin offers deeper tissue penetration due to its longer activation wavelength and shorter duration of skin photosensitivity, making it a more clinically favorable PDT option [102].

Talaporfin sodium (NPe6) is a second-generation chlorin-based PS designed to improve the limitations of first-generation PS such as photofrin. It exhibits strong absorption at ~664 nm, which allows deeper tissue penetration and enhances PDT efficacy in solid tumors. Talaporfin accumulates in tumor tissues after intravenous administration, where activated by red light, undergoing excitation to a triplet state that transfers energy to molecular oxygen. This process generates ^1^O_2_, and other ROS oxidize lipids, proteins, and nucleic acids, leading to apoptosis and necrosis of cancer cells [104]. Talaporfin-PDT induces vascular damage, resulting in vessel occlusion and reduced nutrient supply, thereby enhancing tumor destruction [103]. Compared to photofrin, talaporfin offers shorter drug clearance times, reducing prolonged skin photosensitivity and providing higher Φ_Δ_ due to its chlorin structure [94].

Verteporfin and talaporfin are generally considered better than photofrin because they overcome first-generation limitations, such as long photosensitivity and shallow penetration. Between the two, verteporfin offers deeper penetration at about ~689 nm than talaporfin. However, talaporfin provides high singlet oxygen yield and rapid clearance. The choice depends on tumor type, location, and clinical setting. This is compared with the natural ligand-metal complexes discussed in Section 9.

## 12. Discussion

Coumarin and curcumin are attractive natural ligands for PDT as they combine biocompatibility with intrinsic pharmacological activity. These compounds are derived from traditional Chinese medicinal plants, exhibit antioxidant, anti-inflammatory, and anticancer properties, and synergize with PDT mechanisms [13,14,15,16,17,18,19,20,21,22]. The natural ligand scaffolds improve solubility and reduce systemic toxicity compared to synthetic ligands, which provide moderate ROS generation [23,24]. However, there are some photophysical limitations, such as shallow tissue penetration and relatively low Φ_Δ,_ necessitating enhancement through metal coordination. Ruthenium(II) and Iridium(III) complexes are the choice of collaborators or partners because of their strong spin-orbit coupling, which promotes efficient ISC and high triplet quantum yields [25,26,27,28,29,30,31]. These metals extend absorption into the visible/NIR region, enabling deep tissue penetration and hypoxia-tolerant PDT [32,33,34,35,36,37,38,39,40,41,42,43,44,45,46,47]. Thus, natural ligands and Ru(II)/Ir(III) metal ions form multifunctional, biocompatible PSs to integrate traditional pharmacology with advanced photophysics for the next-generation PDT strategy against cancer. More importantly, natural ligands and Ru(II)/Ir(III) metal ions complement the advantages for PDT and overcome the limitations (Table 10). Many natural ligands can act as PSs; coumarin and curcumin are selected as the central focus for this review because of their well-studied pharmacological activity, photophysical properties, and potential for Ru/Ir coordination.

Compared with free ligands, Ru(II) and Ir(III) complexes coordinated with coumarin or curcumin exhibit markedly enhanced photophysical properties. The coordination extends absorption into the visible and NIR regions, which increases ISC efficiency and raises ΦΔ up to ~0.78 [32,40,44]. These complexes demonstrate strong luminescence and organelle-specific localization, resulting in nanomolar photocytotoxicity under both normoxia and hypoxia [32,44]. Thus, coumarin and curcumin alone have limited PDT potential. The Ru/Ir complexes provide superior photophysical performance and therapeutic efficacy, underscoring the translational relevance of ligand-metal hybrid systems (Table 11).

Table 3 and Table 4 are intentionally included to serve as the necessary baseline benchmarks (control groups) for this review. These conventional Ru/Ir complexes without natural ligands serve as control groups. This becomes fundamental evidence that natural ligands confer significant advantages, including extended absorption into the visible/NIR region, higher Φ_Δ_, and prevention of photocytotoxicity. Thus, the inclusion of these baseline tables strengthens the translational argument by demonstrating the incremental value of coumarin and curcumin coordination.

Natural ligands constitute a diverse but unified class of scaffolds with potential as PSs. Differences in the structure of compounds such as coumarin and curcumin share advantages, including biocompatibility, intrinsic pharmacological activity, and the ability to coordinate transition metals. The limitations include poor solubility, photoinstability, and narrow absorption, and Ru(II)/Ir(III) coordination can provide a strategy for overcoming these drawbacks, thereby positioning natural ligands as versatile scaffolds for designing next-generation PDT agents as described above.

However, coumarin and curcumin have shorter λ_abs_ and λ_em_, lower photostability, and remain at the preclinical stage of development in contrast to the conventional organic PSs (e.g., porphyrins, BODIPY) (Table 12). Despite these photophysical limitations, coumarin and curcumin exhibit intrinsic antioxidant, anti-inflammatory, and anticancer properties that provide multifunctional therapeutic benefits beyond PDT. They are safer, holistic, and more versatile options.

To address the clinical translation challenges of coumarin and curcumin, nanodelivery systems have been developed in recent years to overcome the limitations of shorter λ_abs_ and λ_em_ and lower photostability, but they remain at the preclinical stage. For example, Chaudhuri reported squaric acid-coumarin-chlorambucil: photoresponsive single-component fluorescent organic nanoconjugates for self-monitored therapeutics. The multifunctional squaric acid-coumarin-chlorambucil nanoconjugate was prepared by a simple reprecipitation technique, which exhibited anticancer activity through the synergistic cytotoxic effect of PDT and chemotherapy on HeLa cells, as well as improved therapeutic efficiency for cellular imaging and self-monitoring of the drug release [117]. Kah et al. developed a photoactive curcumin-silver nanoparticle-polymer conjugate to enhance the effectiveness of PDT, as elevated ROS production was induced, leading to apoptosis in lung cancer cells [118].

Another approach is nanoprodrugs, which have improved PDT by packaging light-activated Ir(III) complexes into smart nanoparticles. These systems transport the drug safely through the body and selectively accumulate at tumor sites. They release the active cancer-killing agents only when triggered by specific cues, such as light exposure or the hypoxic tumor microenvironment [45]. It is advantageous to combine chemotherapy with Type I or hypoxia-responsive PDT mechanisms, enabling effective treatment of low-oxygen tumor cores where conventional approaches are limited [119].

In addition, Ru(II) and Ir(III) complexes serve as dual theranostic agents in PDT because they combine potent therapeutic activity with imaging capability. Strong spin-orbit coupling promotes efficient ISC, producing long-lived triplet states that generate ROS for cancer cell destruction. These triplet states produce delayed phosphorescence lifetimes in the microsecond range at the same time, which bypass cellular autofluorescence and enable high-contrast optical imaging. This dual functionality allows simultaneous monitoring of drug localization and therapeutic response to a diagnosis and treatment platform for cancer [25,26,27,28,29,30,31].

Generally, coumarin and curcumin represent two natural ligands with distinct photophysical behaviors in PDT. Coumarin has a low singlet oxygen yield and primarily favors the Type I mechanism, generating radical species through electron transfer, which is more favorable in hypoxic tumor environments but limits its efficacy. Curcumin demonstrates moderate singlet oxygen production and undergoes the Type II mechanism, which is more efficient under normoxic conditions and clinically advantageous for conventional PDT. Hence, curcumin is generally the stronger natural PS, and coumarin’s utility is enhanced through coordination with transition metals, Ru(II), or Ir(III), enabling dual Type I/II activity to improve therapeutic effects (Table 13).

Ruthenium(II) and Iridium(III) complexes have emerged as PSs for PDT that have strong spin-orbit coupling, long-lived triplet states, and efficient ^1^O_2_ generation. Standalone Ru/Ir complexes exhibit potent photocytotoxicity, with IC_50_ values in the nanomolar range, and can induce apoptosis, ferroptosis, as well as ICD under normoxia and hypoxia [32,38,44]. These effects include calreticulin exposure, ATP release, and HMGB1 secretion, which recruit dendritic cells and activate CD8^+^ cytotoxic T-cells, establishing systemic antitumor immunity. However, concerns remain regarding heavy-metal toxicity and limited biocompatibility.

By contrast, natural ligand-metal complexes integrate intrinsic pharmacological activity with enhanced photophysics. Natural ligands buffer heavy-metal toxicity, improve solubility, and provide antioxidant and anticancer properties [13,19]. Coordination with Ru/Ir extends absorption into the near-infrared region, increases Φ_Δ_ up to ~0.78, and enhances hypoxia tolerance [40,41]. These complexes not only achieve localized cytotoxicity but also synergize with immune checkpoint blockade, converting “cold” tumors into “hot” ones responsive to immunotherapy [89,91]. Thus, natural ligand-Ru/Ir complexes represent a next-generation PDT strategy, combining the immune-activating potential of transition metals with the biocompatibility and therapeutic selectivity of plant-derived scaffolds.

Based on comparative data from the tables, clear distinctions are revealed between Ru(II)/Ir(III) complexes coordinated with coumarin versus curcumin. Coumarin-based complexes start from a weaker photophysical baseline, as free coumarin shows limited absorption from ~320 to 380 nm and negligible Φ_Δ_ <0.1 [23]. However, once coordinated with Ru(II) or Ir(III), these complexes achieve enhanced intersystem crossing and red-region phosphorescence, with Φ_Δ_ rising to ~0.67 in polypyridyl derivatives [40]. Biological outcomes include lysosomal and ER localization, induction of apoptosis, ferroptosis, and ICD, with inhibition rates exceeding 90% in lung and breast cancer models [32,38]. Thus, coumarin complexes demonstrate reliable cytotoxicity, though IC_50_ values often remain in the low micromolar range.

Curcumin-derived complexes benefit from stronger intrinsic ligand activity and broader absorption from ~410 to 430 nm, with a higher Φ_Δ_ of ~0.2–0.6 [24]. Coordination with Ru(II)/Ir(III) further extends absorption into the NIR region, producing nanomolar IC_50_ values in resistant cancer cells and 3D spheroids [36,44]. These complexes indicate mitochondrial targeting, ferroptosis induction, and synergy with chemotherapy, while nanocarrier encapsulation improves solubility and tumor selectivity [45,48]. The curcumin systems consistently demonstrate potent ICD induction and translational promise, with photocytotoxicity as low as 0.012 µM under hypoxia [44].

Coumarin complexes provide valuable organelle specificity and immune activation, but curcumin-derived Ru/Ir complexes outperform in photophysical efficiency, cytotoxic potency, and translational relevance for clinical PDT development [24,36,40,44,45,48].

Clinical FDA-approved PS agents in PDT such as photofrin, verteporfin, and talaporfin are effective in localized tumor ablation, but they suffer from drawbacks, such as prolonged photosensitivity, limited tissue penetration, and reduced efficacy under hypoxia. Thus, Ru(II) and Ir(III) complexes coordinated with natural ligands demonstrate superior photophysical properties, including near-infrared absorption, high Φ_Δ_ (up to 0.78), and hypoxia tolerance [40,44]. These complexes induce ICD, recruiting dendritic cells and activating CD8^+^ T-cells, thereby achieving systemic antitumor immunity, which is an effect not typically observed with clinical PSs. For instance, Ru(II) polypyridyl complexes suppressed colorectal tumors while synergizing with checkpoint blockade [89], whereas Ir(III) complexes disrupted mitochondrial respiration to amplify ROS and immune activation [91]. Compared to clinical PSs, natural ligand-metal complexes offer dual benefits, including intrinsic pharmacological activity from coumarin or curcumin and advanced immune modulation from Ru/Ir centers. This positions them as next-generation PSs in PDT capable of overcoming hypoxia, enhancing tumor selectivity, and integrating with immunotherapy, thereby extending PDT beyond local cytotoxicity and toward durable systemic oncology outcomes.

However, clinical approval requires rigorous evaluation of long-term safety, pharmacokinetics, and heavy-metal toxicity. Natural ligands buffer biocompatibility and reduce systemic toxicity [13,19]; Ru and Ir remain rare heavy metals with potential accumulation risks. Compared to FDA-approved PSs like photofrin or verteporfin, natural ligand-metal complexes are still at the preclinical stage. Thus, they can achieve clinical approval if future translational studies confirm safety, efficacy, and scalable nanodelivery systems, but they cannot be considered clinically approved PSs.

There is an important safety concern in the development of Ru(II) and Ir(III) complexes for PDT that accumulate in tissues, leading to off-target cytotoxicity and long-term adverse effects [10]. Rational ligand design using biocompatible scaffolds such as coumarin or curcumin mitigates these risks and maintains therapeutic efficacy [13,14,15,16,17,18,19,20,21,22]. Biodegradable nanocarriers further enhance safety by improving solubility, controlling biodistribution, and enabling targeted release at tumor sites [41,45]. Targeted delivery systems, including peptide conjugates and nanoprodrugs, provide spatiotemporal control, reducing systemic exposure and collateral damage. These strategies balance efficacy with safety, supporting the clinical translation of natural ligand-metal complexes in PDT [11,45].

Coumarin-based metal complexes are more widely studied in PDT than curcumin complexes, because the former offer better photophysical properties (higher stability, tunable fluorescence, and easier coordination with metals), while curcumin suffers from poor stability, short **λ_abs_**, and lower Φ_Δ_, as illustrated in Table 10. Meaanwhile, curcumin is still more well-known than coumarin because of its strong cultural roots, widespread dietary use, and broad pharmacological activities. As the active compound in turmeric, curcumin has been consumed for centuries in traditional medicine and food, a concept called “medicine and food homology”, making it highly recognizable and safe at dietary levels [120]. Its diverse biological and pharmacological effects, including anti-inflammatory, antioxidant, and anticancer properties, have been supported by thousands of studies and clinical trials, reinforcing its global reputation [121]. In contrast, coumarin is restricted in consideration of hepatotoxicity and remains a niche scaffold in PDT research, despite superior photophysical properties [122].

## 13. Conclusions

PDT has advanced significantly through the development of diverse PSs, and the clinical translation remains challenging for natural ligands such as coumarin and curcumin, although they have intrinsic properties, such as antioxidant, anti-inflammatory, and anticancer multifunctional therapeutic effects. The limitations in absorption and emission ranges, photostability, and Φ_Δ_ restrict their clinical study. Recent progress in nanodelivery systems has addressed the corresponding challenges.

Natural ligand-metal complexes represent a promising strategy to combine the bioactivity of natural ligands with the superior photophysical properties of transition metals such as Ru and Ir. These complexes exploit the heavy-atom effect to enhance ISC, extend absorption into the NIR region, and maintain activity under hypoxic conditions. Heavy-metal toxicity remains, and it must be accounted for in ligand design to mitigate risks through delivery systems.

These Ru/Ir natural ligand complexes not only achieve potent cytotoxicity but also stimulate immunogenic cell death, thereby extending PDT efficacy toward systemic antitumor immunity. Compared with current FDA-approved photosensitizers such as photofrin and verteporfin, they demonstrate superior hypoxia tolerance and immune activation, underscoring their potential as next-generation clinical PSs in PDT. However, they still require rigorous validation in translational animal models and standardized toxicity profiling to ensure safety. Comprehensive pharmacokinetic studies and alignment with regulatory frameworks are essential before these natural ligand-metal complexes can progress from promising preclinical findings to approved clinical use.

Nanotechnology-integrated natural ligand-metal complexes are viable pathways to eliminate barriers to natural ligand-based PDT as a versatile and safer approach for cancer therapy, which can probably integrate with artificial intelligence (AI)-assisted molecular design in the future.

Recent preclinical studies have provided compelling evidence for the translational potential of Ru(II) and Ir(III) complexes in PDT. Gong et al. reported that a Ru(II) complex induced photodynamically triggered ICD in HeLa cells, characterized by calreticulin exposure, ATP release, and HMGB1 secretion, thereby priming CD8^+^ T-cell responses and extending PDT efficacy toward systemic antitumor immunity [88]. Multifunctional nanoplatforms incorporating Ru/Ir complexes have been engineered to enhance solubility, biodistribution, and tumor selectivity, achieving synergistic cancer therapy in vivo [1]. These findings highlight that Ru/Ir complexes can overcome limitations of current FDA-approved photosensitizers, particularly in hypoxic or resistant tumors. Clinical applications may involve combining Ru/Ir complexes with immune checkpoint inhibitors or integrating them into theranostic nanocarriers for simultaneous imaging and therapy. Rigorous pharmacokinetic profiling and translational animal models remain essential to bridge preclinical promise with clinical adoption.

Looking forward, future perspectives emphasize rational ligand engineering, integration with nanotechnology for targeted delivery, and combination strategies with immunotherapy to maximize systemic antitumor effects. AI-assisted molecular design and patient-specific predictive models will further accelerate clinical translation, ensuring that natural ligand-metal complexes evolve from promising laboratory findings into safe, effective, and personalized PDT platforms.

## 14. Future Aspects

Currently, AI is increasingly transforming PDT by streamlining ligand design, nanomedicine optimization, and theranostic integration. Machine learning (ML) models predict photophysical parameters such as Φ_Δ_ and ISC efficiency, guiding the rational design of Ru(II) and Ir(III) complexes with natural ligands [123]. This can support the development of biodegradable carriers and nanoprodrugs, improving solubility, biodistribution, and tumor targeting to reduce systemic toxicity [124]. This enhances phosphorescence signal analysis, enabling simultaneous imaging and therapy for precise treatment monitoring in theranostics [125]. These approaches reduce trial and error in the designation and usage of PS, accelerating the transition from preclinical studies to clinical translation.

Beyond molecular design, AI can support personalized PDT by integrating patient-specific genomic and immunological data to predict therapeutic response. It may further optimize combination strategies with immune checkpoint inhibitors, enhancing systemic antitumor immunity. AI-driven PDT development must align with regulatory standards and ethical frameworks to ensure reproducibility, safety, and clinical acceptance.

Thus, AI-assisted strategies promise safer [126], multifunctional PDT agents to integrate natural bioactivity with advanced photophysics, positioning ligand-metal complexes as versatile platforms for cancer therapy.

## Figures and Tables

**Table 1:** Natural Ligands from TCM for sources, concepts, and pharmacological activities.

Natural Ligand	Source	TCM Theory	Intrinsic Pharmacological Activities
Coumarin	*Peucedanum decursivum* (Miq.) Maxim (Qián Hú) [13]	“Clear heat”, “detoxification”, “activate blood & remove stasis”, and “dissolve phlegm & mass” concepts align with modern pharmacological outcomes of reducing oxidative stress, inflammation, and cancer progression [14]	Antioxidant Scavenge ROS, such as DPPH and hydrogen peroxide, which is explained by favorable HOMO-LUMO electronic transitions for stabilizing radicals [15] Anti-inflammatory Suppress pro-inflammatory mediators such as nitric oxide (NO), prostaglandin E2 (PGE2), and cytokines through inhibition of NF-κB and MAPK signaling pathways to reduce inflammation and restore balance [16] Anticancer Modulate tumor-immune interactions and suppress oncogenic signaling pathways by inhibition of NF-κB and STAT3 activation, reduce pro-tumor cytokines, and enhance cytotoxic T-cell responses, promote apoptosis and arrest cancer cells cycle [17]
Curcumin	Dried *rhizome* of *Curcuma longa* L. [18]	“Clear heat”, “detoxification”, and “resolve toxicity” concepts align with modern pharmacological outcomes of reducing oxidative stress, maintaining systemic balance, alleviating inflammatory disorders, and restraining tumor progression [19]	Antioxidant Scavenges free radicals, upregulates antioxidant enzymes such as superoxide dismutase and catalase, and protects cellular components from oxidative damage [20] Anti-inflammatory Modulate signaling pathways such as NF-κB, MAPK, and JAK/STAT activation to reduce the expression of pro-inflammatory cytokines (TNF-α, IL-6, IL-1β) and enzymes like COX-2 and iNOS to suppress oxidative stress induced inflammation and regulate immune responses [21] Anticancer Suppress sodium-dependent vitamin C transporter 2 (SVCT2) expression to reduce intracellular vitamin C uptake for cancer cell proliferation and metastasis [22]

**Table 2:** Chemical structure and photophysical features of natural ligands (PS).

Natural Ligands (PS)	Chemical Structure	Photophysical Features	
Coumarin [23]	Benzopyrone scaffold 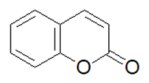	Absorption (λ_abs_): ~320–380 nm (peak ~350 nm) Excitation (λ_ex_): ~340–360 nm Emission (λ_em_): ~400–480 nm (blue fluorescence) Singlet oxygen quantum yield (Φ_Δ_): <0.1	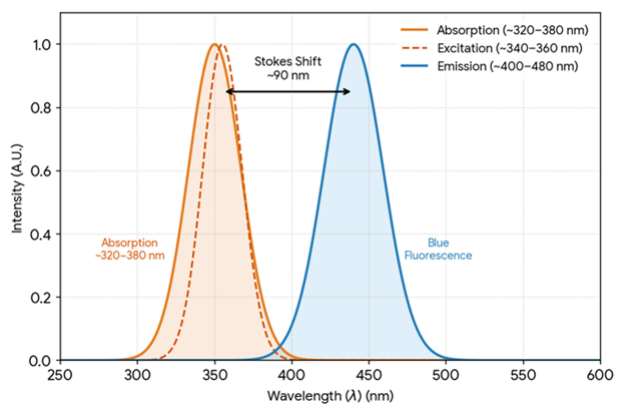
Curcumin [24]	Polyphenolic β-diketone (keto–enol tautomerism) 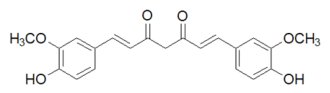	Absorption (λ_abs_): ~410–430 nm Excitation (λ_ex_): ~420 nm Emission (λ_em_): ~460–550 nm Singlet oxygen quantum yield (Φ_Δ_): ~0.2–0.6	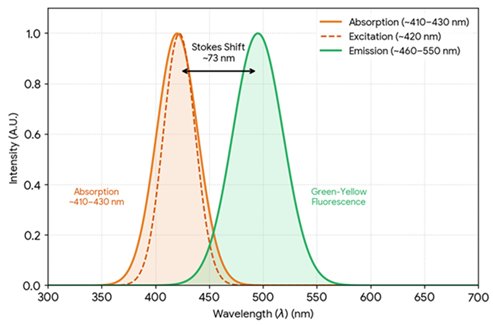

**Table 3:** Past studies on coumarin in PDT against cancer.

Topic	Metal Complex & Photophysical Features	Consequence
COUPY coumarins as novel mitochondria-targeted photodynamic therapy anticancer agents [54]	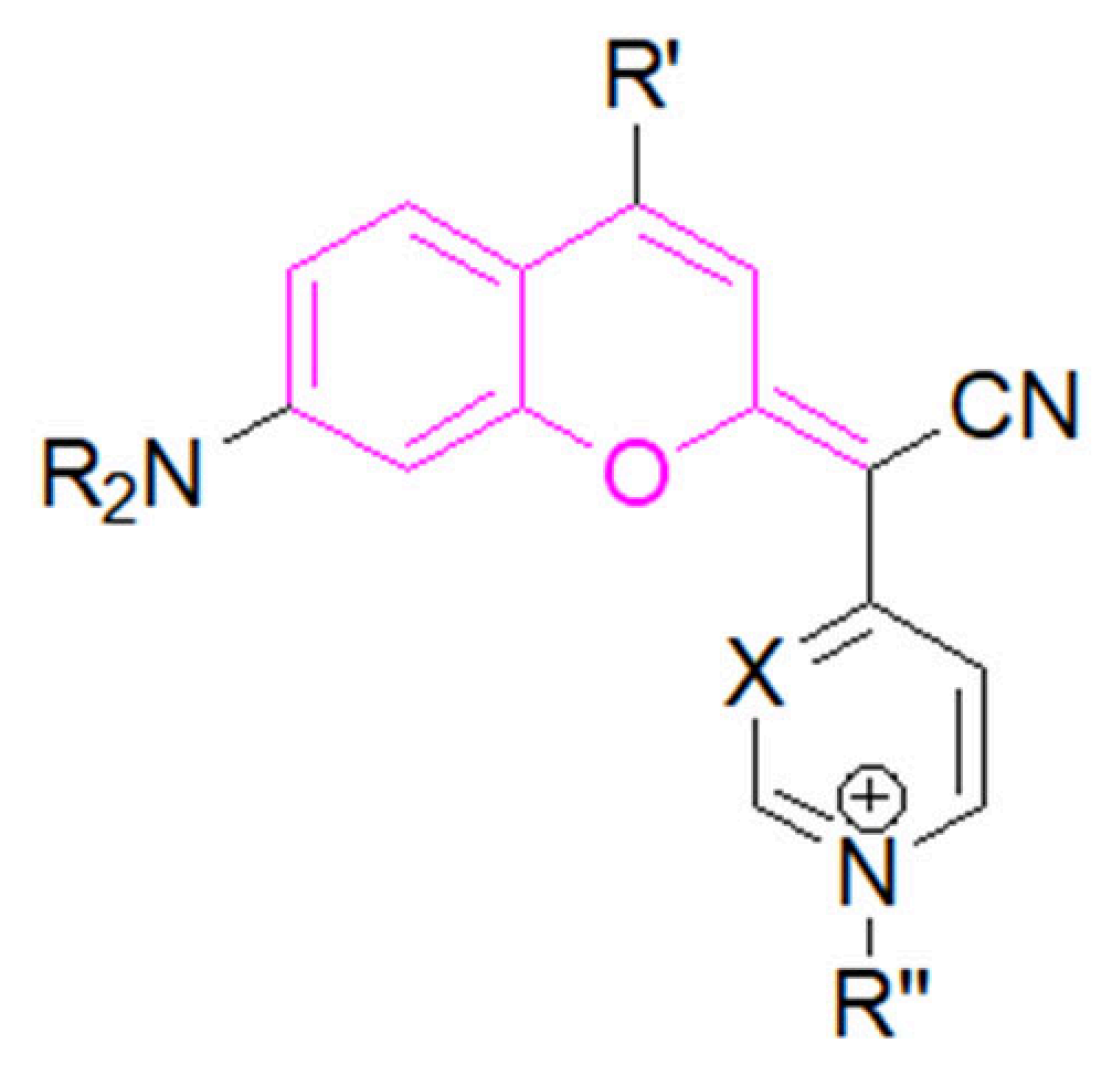 **IC_50_** (µM, **HeLa**) for Dark: not numerically specified, Light: 0.19 ± 0.03 **IC_50_** (µM, **A2780**) for Dark: not numerically specified, Light: 0.09 ± 0.02 **IC_50_** (µM, **CHO**) for Dark: not numerically specified, Light: 1.4 ± 0.3 **PDT light dose**: 420–520 nm (visible-light irradiation) **Excitation (λ_ex_)**: ~520 nm (MLCT/coumarin band) **Absorption (λ_abs_)**: 400–600 nm (broad visible absorption) **Absorption maximum (λ_abs max_)**: ~510–520 nm (Q-band region) **Emission (λ_em_)**: ~600–620 nm (red phosphorescence) **Singlet oxygen quantum yield (Φ_Δ_)**: 0.11	COUPY coumarin was the theranostic agent targeting mitochondria to generate the cytotoxic ROS for the induction of apoptosis and or autophagy after irradiation with low doses of visible light
Improving photodynamic therapy anticancer activity of a mitochondria-targeted coumarin photosensitizer using a polyurethane-polyurea hybrid nanocarrier biomacromolecules [55]	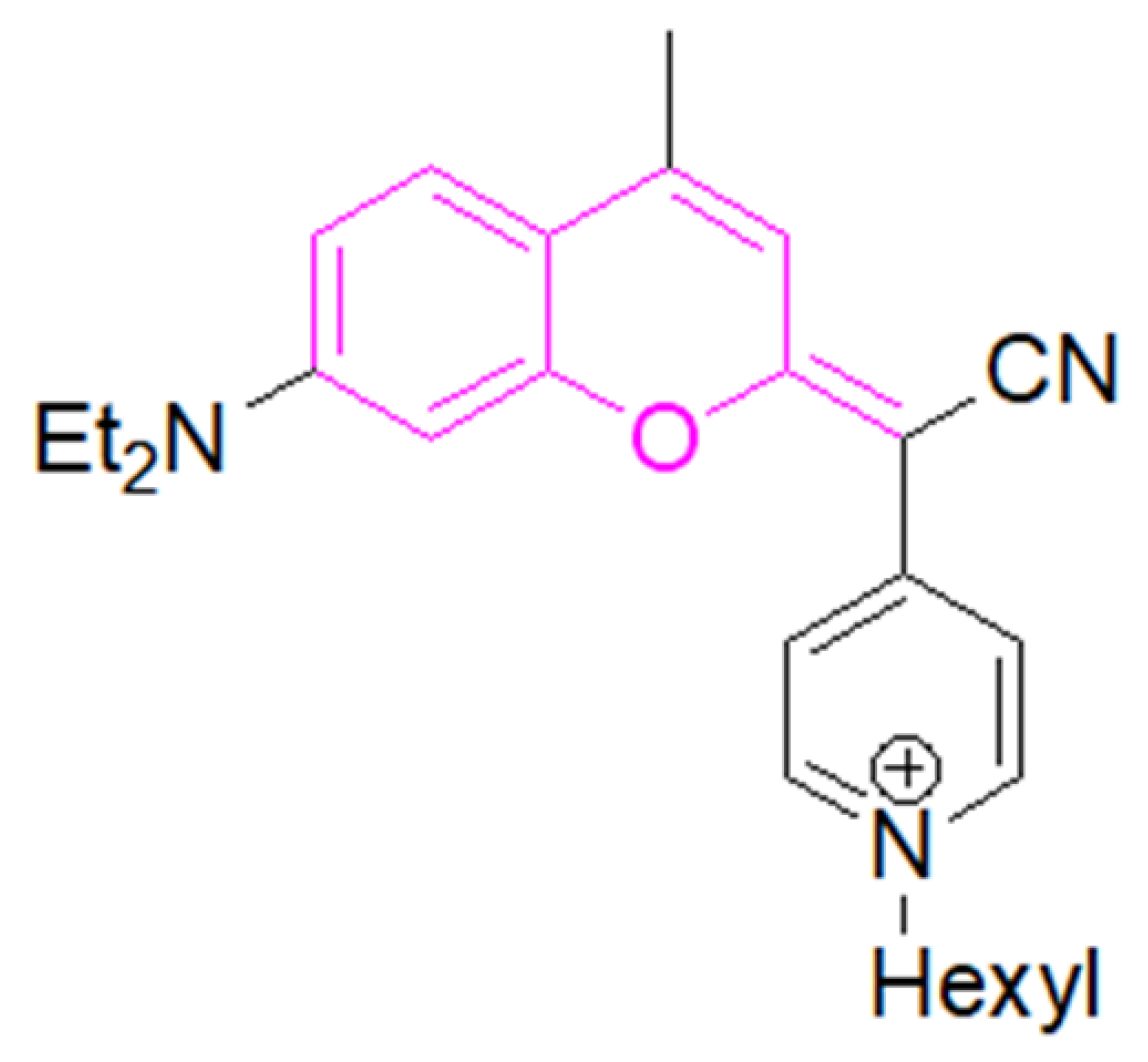 **IC_50_** (µM, **HeLa**) for Dark: 199 ± 14, Light: 0.78 ± 0.09 **IC_50_** (µM, **A2780 cis**) for Dark: 20 ± 2, Light: 0.7 ± 0.1 **PDT light dose**: 520 nm, 89 mW/cm^2^, 1 h **Excitation (λ_ex_)**: 520 nm (green light excitation) **Absorption (λ_abs_)**: 400–600 nm (broad visible absorption) **Absorption maximum (λ_abs max_)**: ~510–520 nm (Q-band region) **Emission (λ_em_)**: ~600–620 nm (red phosphorescence) **Singlet oxygen quantum yield (Φ_Δ_)**: 0.0076	Nanoencapsulation decreased dark cytotoxicity of the coumarin PS and significantly improved in vitro photoactivity with red light toward cancer cells, and induced mitochondrial photodamage with ROS photogeneration to trigger autophagy and apoptotic cell death of cancer cells
A dual action coumarin-camptothecin polymer for light responsive drug release and photodynamic therapy [56]	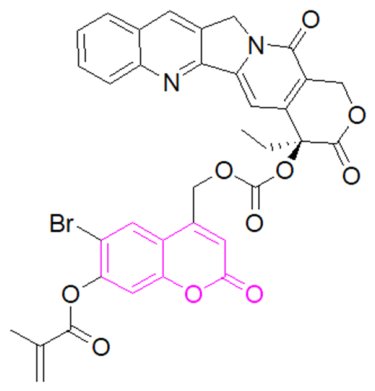 **IC_50_** (µM, **HeLa**) for Dark: 100, Light: 0.19 ± 0.03 **PDT light dose**: 365 nm, 15 mW/cm^2^, 2 min **Excitation (λ_ex_)**: 365 nm **Absorption (λ_abs_)**: 300–400 nm (coumarin absorption region) **Absorption maximum (λ_abs max_)**: ~365 nm **Emission (λ_em_)**: ~450–500 nm (blue fluorescence) **Singlet oxygen quantum yield (Φ_Δ_)**: 0.44	Coumarin-camptothecin polymer demonstrated good biocompatibility in the dark and high cancer cell killing activity mediated by the photo-release of camptothecin and ROS generation in PDT
Calixarene-based nanostructures for delivering coumarin 6 for tumor-cell imaging and photoinduced toxicity [57]	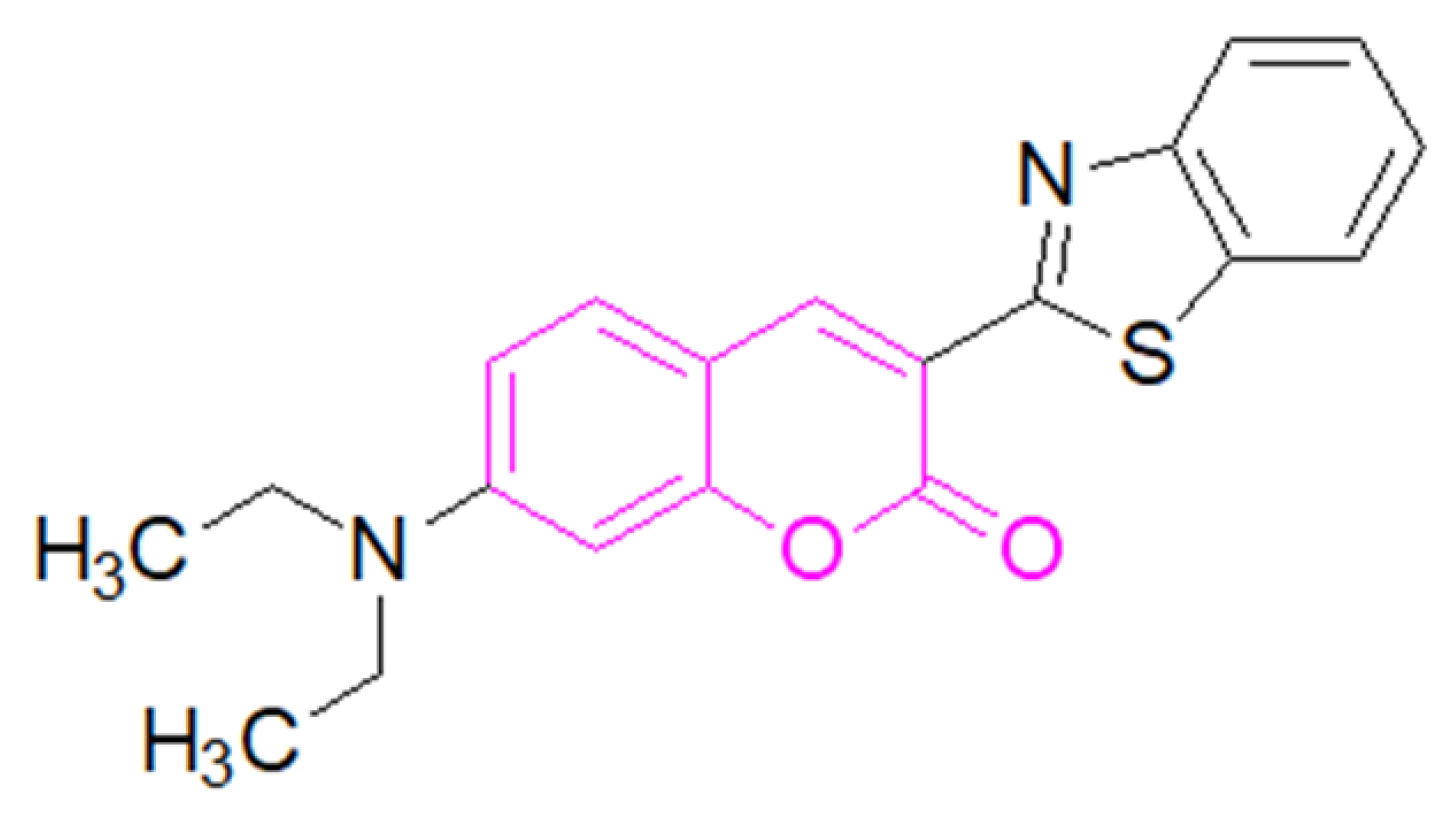 **IC_50_** (µM, **Hep3B**) for Dark: >100, Light: 12.3 ± 1.5 **IC_50_** (µM, **MCF-7**) for Dark: >100, Light: 10.8 ± 1.2 **PDT light dose**: 470 nm, 800 mW/cm^2^, 15 min **Excitation (λ_ex_)**: 470 nm (coumarin absorption region) **Absorption (λ_abs_)**: 400–500 nm (broad visible absorption) **Absorption maximum (λ_abs max_)**: 470 nm **Emission (λ_em_)**: 507 nm (green fluorescence) **Singlet oxygen quantum yield (Φ_Δ_)**: 0.44	Fluorescent nanosystem obtained by entrapping coumarin 6 in biocompatible nanostructures formed through the self-assembly of an amphiphilic calix[4]arene derivative functionalized with choline ligands for targeting tumor cells
Modulation of the photodynamic activity of a cinnamoyl-coumarin-RGD peptide conjugate via cucurbit [8] uril supramolecular assembly [58]	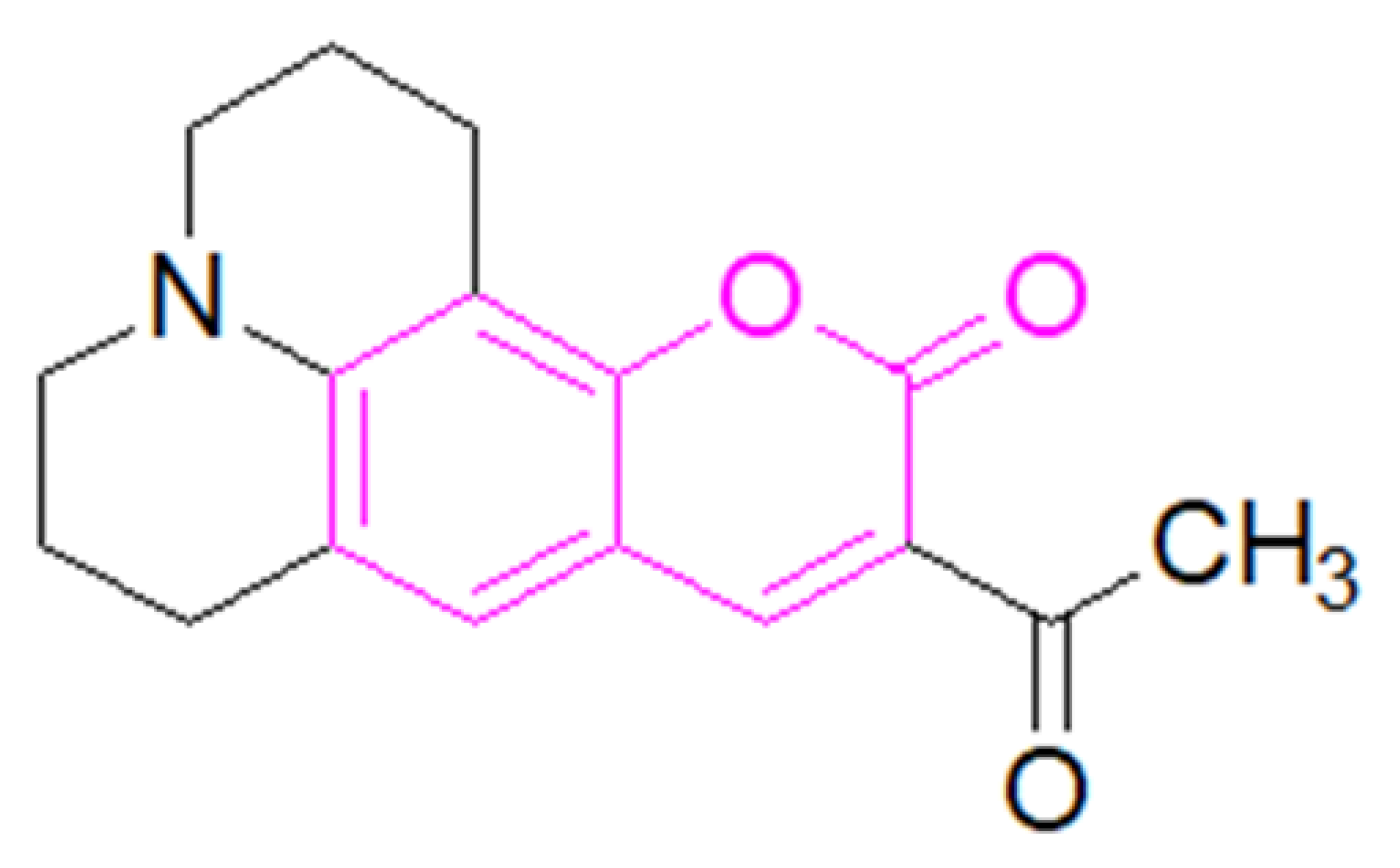 **IC_50_** (µM, **MCF-7**) for Dark: >100, Light: 0.42 ± 0.05 **PDT light dose**: 450 nm, 250 mW/cm^2^, 1 min (15 J/cm^2^) **Excitation (λ_ex_)**: 450 nm (coumarin absorption region) **Absorption (λ_abs_)**: 350–500 nm (broad visible absorption) **Absorption maximum (λ_abs max_)**: ≈450 nm **Emission (λ_em_)**: ≈520 nm (green fluorescence) **Singlet oxygen quantum yield (Φ_Δ_)**: 0.62	Cinnamoyl-coumarin-RGD peptide conjugate (PS-GG(KG)_3_G-RGD) demonstrated tumor targeting capabilities, improved aqueous solubility, and photodynamic activity in MCF-7 modulation by cucurbit [8] uril (CB [8]) complexation
Targeted photoresponsive TiO_2_-coumarin nanoconjugate for efficient combination therapy in MDA-MB-231 breast cancer cells: synergic effect of photodynamic therapy (PDT) and anticancer drug chlorambucil [59]	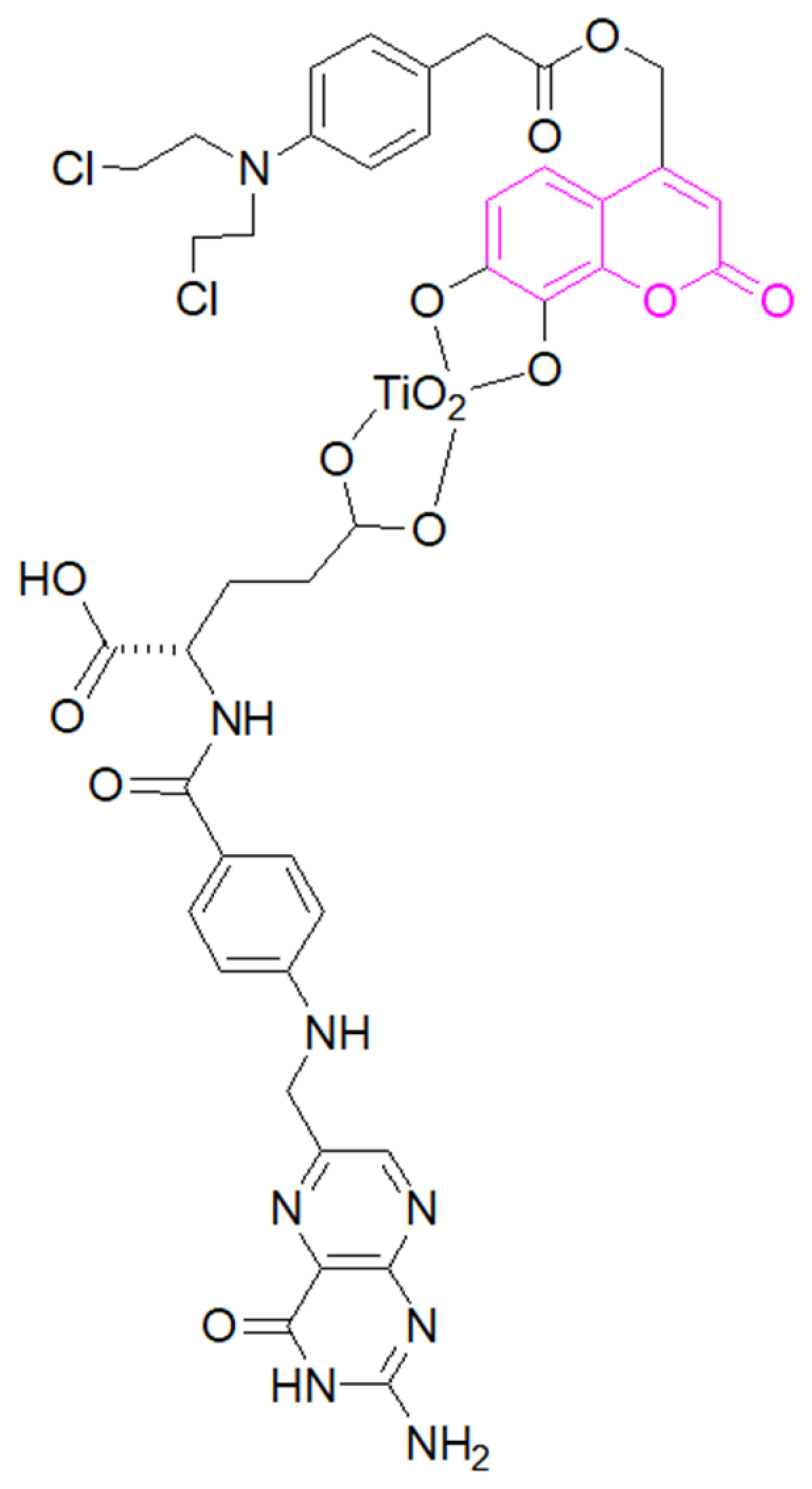 **IC_50_** (µM, **MDA-MB-231**) for Dark: >100, Light: ~0.42–0.78 **PDT light dose**: ≥410 nm, 125 mW/cm^2^, 160 min **Excitation (λ_ex_)**: 360 nm **Absorption (λ_abs_)**: 300–600 nm (broad visible absorption) **Absorption maximum (λ_abs max_)**: ~360–410 nm (coumarin absorption region) **Emission (λ_em_)**: ~450–500 nm (blue fluorescence) **Singlet oxygen quantum yield (Φ_Δ_)**: exact value of the Φ_Δ_ was not provided, but ROS generation was observed	TiO_2_-coumarin nanoconjugate significantly inhibited tumor cell proliferation and increased induction of apoptosis, which was benign to the physiological system, and exhibited high therapeutic efficacy
Targeted photoresponsive carbazole-coumarin and drug conjugates for efficient combination therapy in leukemia cancer cells [60]	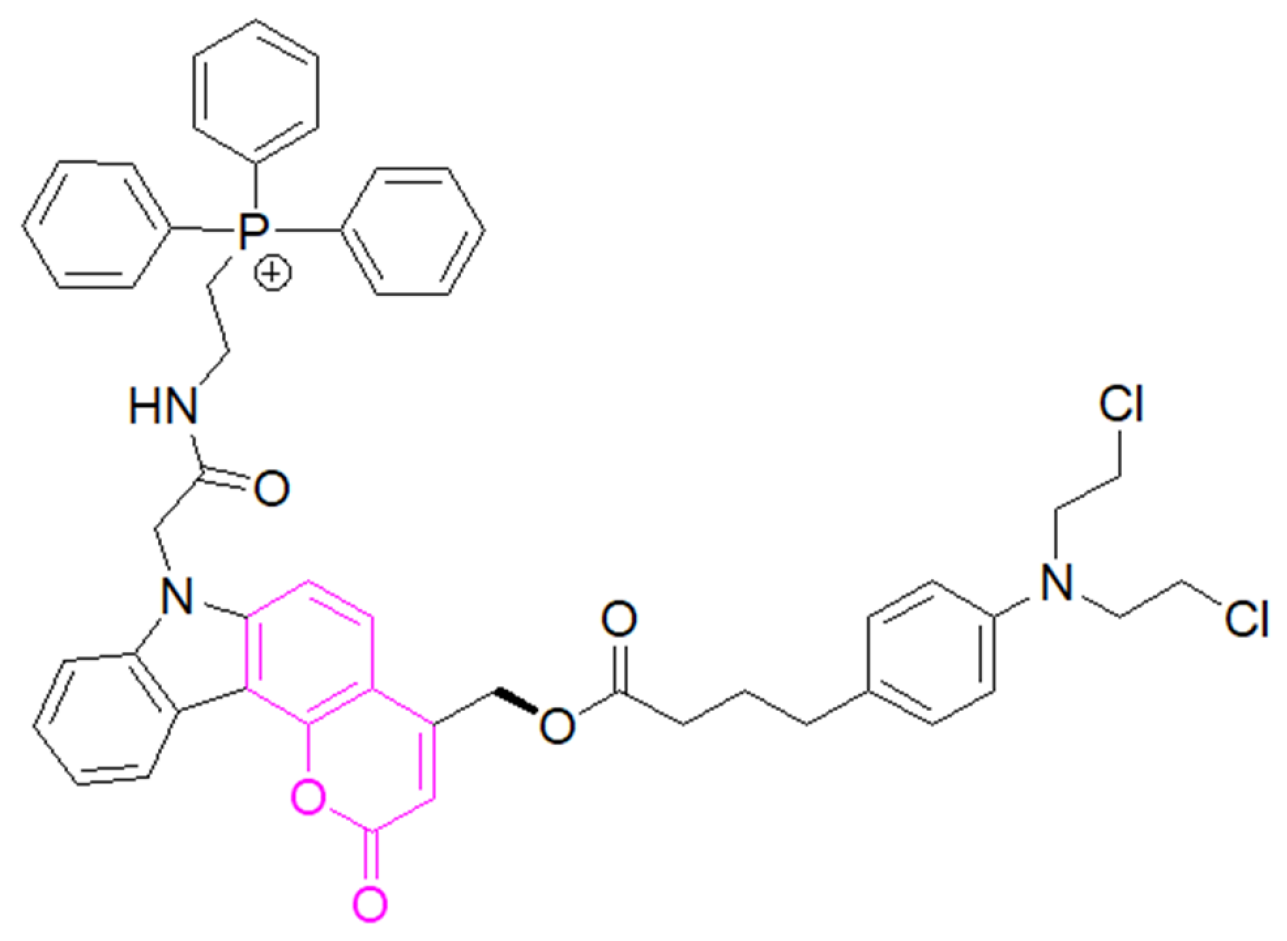 **IC_50_** (µM, **CC-7**) for Dark: >80, Light: 0.8 **IC_50_** (µM, **CC-8**) for Dark: >80, Light: 0.1 **PDT light dose**: ≥405 nm (laser diode irradiation) **Excitation (λ_ex_)**: 405 nm **Absorption (λ_abs_)**: 350–500 nm **Absorption maximum (λ_abs max_)**: ~360 **Emission (λ_em_)**: ~450 (blue fluorescence) **Singlet oxygen quantum yield (Φ_Δ_)**: exact value of the Φ_Δ_ was not provided, but ROS generation was observed	Carbazole-coumarin drug conjugates demonstrated significant suppression of leukemia cell proliferation by targeting mitochondria through the combination therapy

**Table 4:** Past studies on curcumin in PDT against cancer.

Topic	Metal Complex & Photophysical Features	Consequence
Effects of curcumin-based PDT on the viability and the organization of actin in melanotic (A375) and amelanotic melanoma (C32) *in vitro* studies [61]	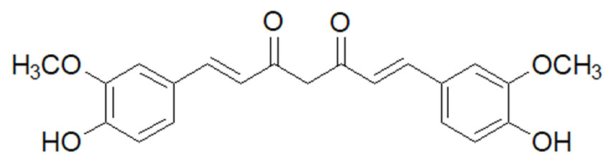 **IC_50_** (µM, **A375**) for Dark: ~50–100, Light: 10.5 ± 1.3 **IC_50_** (µM, **C32**) for Dark: ~50–100, Light: 9.2 ± 1.1 **IC_50_** (µM, **HaCaT**) for Dark: ~25–50, Light: 8.7 ± 1.0 **IC_50_** (µM, **HGF**) for Dark: ~25–50, Light: 8.7 ± 1.0 **PDT light dose**: 410 nm, 20 mW/cm^2^, 5 min (6 J/cm^2^) **Excitation (λ_ex_)**: 410 nm **Absorption (λ_abs_)**: 380–500 nm **Absorption maximum (λ_abs max_)**: 440 nm **Emission (λ_em_)**: ~500–550 nm (green-yellow fluorescence) **Singlet oxygen quantum yield (Φ_Δ_)**: exact value of the Φ_Δ_ was not provided, but ROS generation was observed	Curcumin-PDT reduced melanoma cell viability, disrupted the actin cytoskeleton, and induced apoptosis through ROS generation, indicating strong anticancer potential but limited selectivity toward normal skin cells
Evaluation of curcumin-mediated photodynamic therapy on the reverse of multidrug resistance in tumor cells [62]	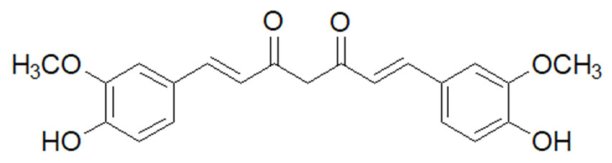 **IC_50_** (µM, **MCF-7**) for Dark: >100, Light: ~7.5 ± 0.8 **IC_50_** (µM, **MCF-7/ADM**) for Dark: >100, Light: ~8.5 ± 0.9 **PDT light dose**: 450 nm, 100 mW/cm^2^, 5 min **Excitation (λ_ex_)**: 450 nm **Absorption (λ_abs_)**: 400–500 nm **Absorption maximum (λ_abs max_)**: ~440–450 nm **Emission (λ_em_)**: ~500–550 nm (green-yellow fluorescence) **Singlet oxygen quantum yield (Φ_Δ_)**: exact value of the Φ_Δ_ was not provided, but ROS generation was observed	Curcumin-PDT induced apoptosis in resistant MCF-7/ADM cells primarily through the endogenous mitochondrial apoptosis pathway, which induced apoptosis activation in resistant cells and the reverse of multidrug resistance for the downregulation of P-gp expression
Assessing the effects of curcumin and 450 nm photodynamic therapy on oxidative metabolism and cell cycle in head and neck squamous cell carcinoma: an *in vitro* study [63]	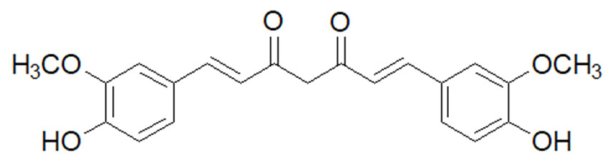 **IC_50_** (µM, **OHSU-974**) for Dark: >100, Light: ~8.7 ± 1.0 **PDT light dose**: 450 nm, 250 mW/cm^2^, 1 min (15 J/cm^2^) (diode laser, continuous wave mode) **Excitation (λ_ex_)**: 450 nm **Absorption (λ_abs_)**: 400–500 nm (broad visible absorption) **Absorption maximum (λ_abs max_)**: ~440–450 nm **Emission (λ_em_)**: ~500–550 nm (green-yellow fluorescence) **Singlet oxygen quantum yield (Φ_Δ_)**: exact value of the Φ_Δ_ was not provided, but ROS generation was observed	Curcumin-PDT caused an oxidative phosphorylation metabolism impairment and induced the uncoupling between respiration and energy production, leading to oxidative damage, cellular growth and viability reduction, as well as cell cycle block in the G1 phase for an anticancer effect
Treatment of breast cancer *in vivo* by dual photodynamic and photothermal approaches with the aid of curcumin photosensitizer and magnetic nanoparticles [64]	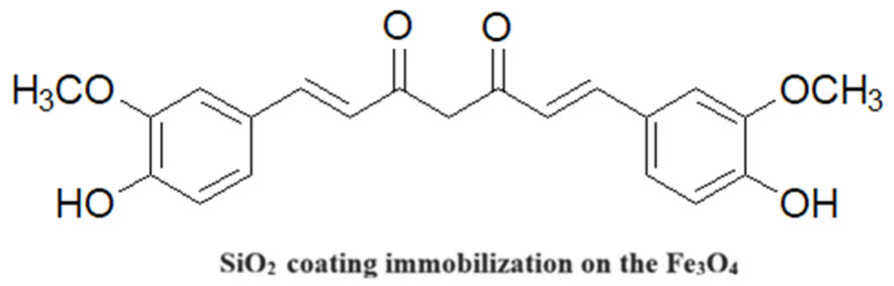 **IC_50_** (µM, **4T1**) for Dark: >100, Light: ~9.8 ± 1.1 **PDT light dose**: 450 nm, 150 mW/cm^2^, 3 min (≈27 J/cm^2^) **Excitation (λ_ex_)**: 435–450 nm **Absorption (λ_abs_)**: 400–500 nm (broad visible absorption) **Absorption maximum (λ_abs max_)**: 435 nm **Emission (λ_em_)**: ~500–550 nm (green-yellow fluorescence) **Singlet oxygen quantum yield (Φ_Δ_)**: exact value of the Φ_Δ_ was not provided, but ROS generation was observed	Fe_3_O_4_/SiO_2_-curcumin nanocomposites expressed the proapoptotic Bax and caspase-3 proteins for the indication of apoptosis for triple negative breast cancers
Effect of curcumin nanoemulsion associated with photodynamic therapy in cervical carcinoma cell lines [65]	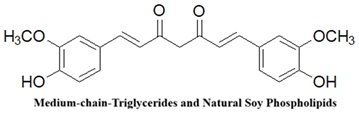 **IC_50_** (µM, **CasKi**) for Dark: ~40–50, Light: ~12 ± 2 **IC_50_** (µM, **SiHa**) for Dark: ~35–45, Light: ~10 ± 1.5 **IC_50_** (µM, **HaCaT**) for Dark: ~60–70, Light: ~25 ± 3 **PDT light dose**: 410 nm LED, 20 mW/cm^2^, 5 min (≈6 J/cm^2^) **Excitation (λ_ex_)**: 410 nm **Absorption (λ_abs_)**: 380–500 nm **Absorption maximum (λ_abs max_)**: ~440 nm **Emission (λ_em_)**: ~500–550 nm (green-yellow fluorescence) **Singlet oxygen quantum yield (Φ_Δ_)**: exact value of the Φ_Δ_ was not provided, but ROS generation was observed in the DCF assay, and mitochondrial disruption	Curcumin nanoemulsion formulation acted as an alternative treatment to cervical lesions using an endoscopic diode fiber laser setup for in situ activation or cavity activation using a diffuse fiber delivery system to increase caspase-3/caspase-7 activities
Photodynamic effect of curcumin on NPC/CNE2 cells [66]	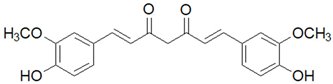 **IC_50_** (µM, **NPC/CNE2**) for Dark: ~16, Light: not numerically specified but significant photocytotoxicity observed **PDT light dose**: 300–400 nm, 60 mW/cm^2^ **Excitation (λ_ex_)**: ~300–400 nm **Absorption (λ_abs_)**: 380–500 nm **Absorption maximum (λ_abs max_)**: ~440 nm **Emission (λ_em_)**: ~500–550 nm (green-yellow fluorescence) **Singlet oxygen quantum yield (Φ_Δ_)**: exact value of the Φ_Δ_ was not provided, but ROS generation was observed in apoptosis, caspase-3 activation, and DNA fragmentation	Curcumin-PDT demonstrated dark cytotoxicity and photocytotoxicity to NPC/CNE2 cells, which induced apoptosis with caspase-3/7 activation
EGFR-targeted photodynamic therapy by curcumin-encapsulated chitosan/TPP nanoparticles [67]	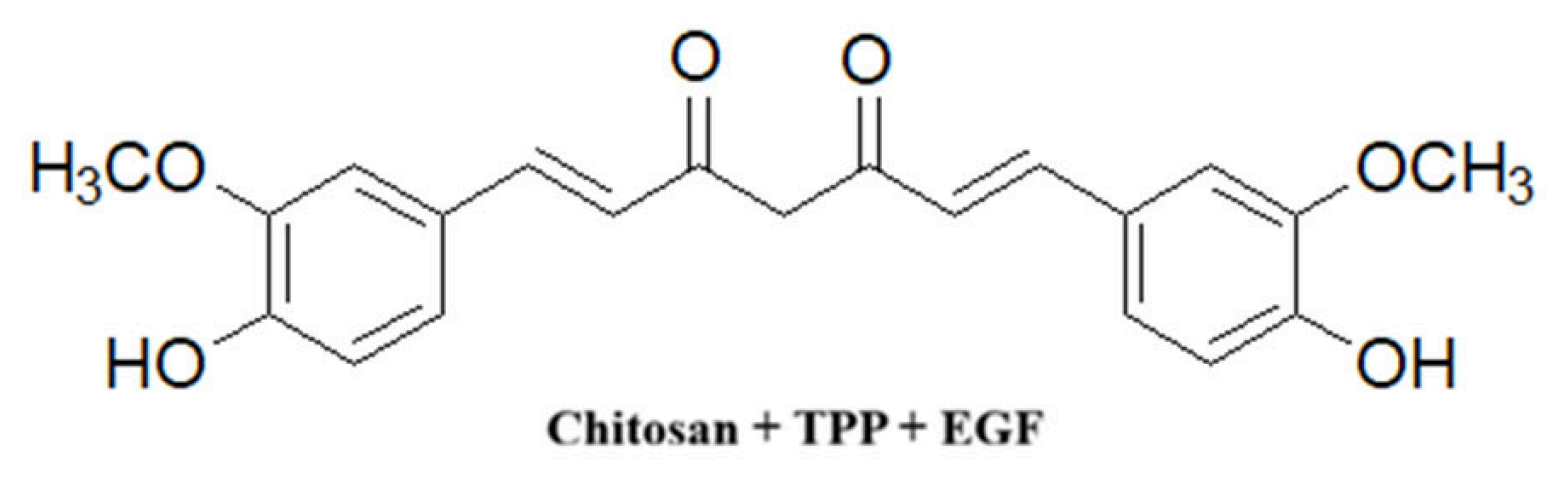 **IC_50_** (µM, **MKN-45**) for Dark: 45 ± 5, Light: 11 ± 2 **IC_50_** (µM, **GES-1**) for Dark: 65 ± 6, Light: 27 ± 3 **PDT light dose**: ~450 nm, 20 mW/cm^2^, 10 min (12 J/cm^2^) **Excitation (λ_ex_)**: ~450 nm **Absorption (λ_abs_)**: 380–500 nm **Absorption maximum (λ_abs max_)**: ~440 nm **Emission (λ_em_)**: ~500–550 nm (green-yellow fluorescence) **Singlet oxygen quantum yield (Φ_Δ_)**: exact value of the Φ_Δ_ was not provided, but ROS generation was observed	Curcumin-encapsulated chitosan/TPP nanoparticles targeted PDT with a specific light source to produce ROS against EGFR-overexpressing cancers
Photodynamic therapy of ovarian carcinoma cells with curcumin-loaded biodegradable polymeric nanoparticles [68]	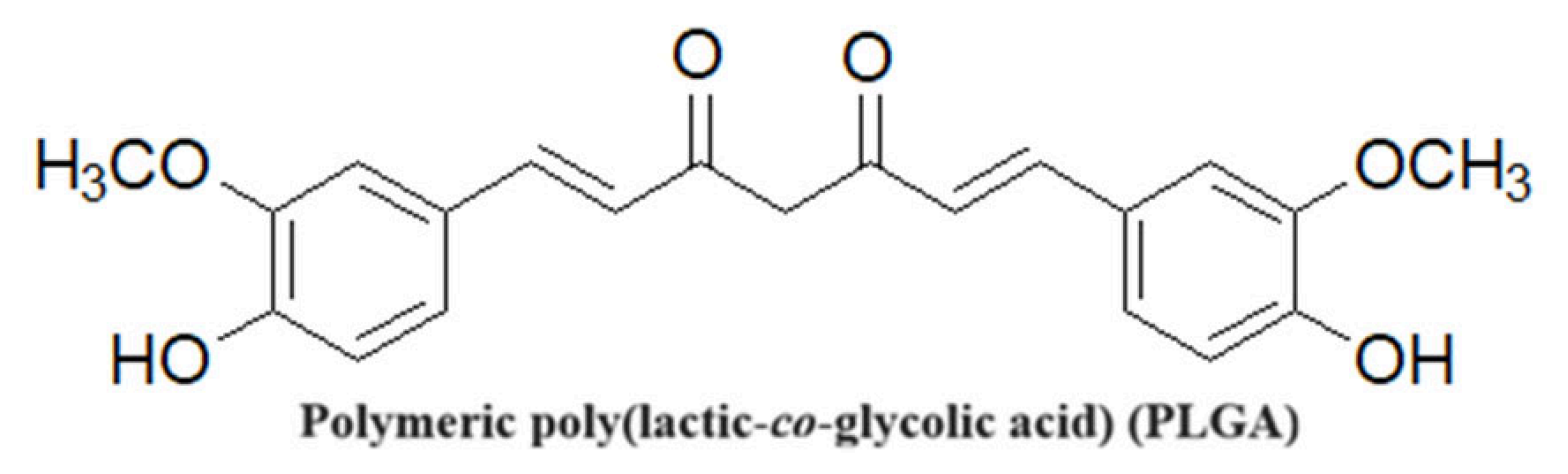 **IC_50_** (µM, **SK-OV-3**) for Dark: 42 ± 4, Light: 11 ± 2 **PDT light dose**: ~450 nm, 20 mW/cm^2^, 10 min (12 J/cm^2^) **Excitation (λ_ex_)**: ~450 nm **Absorption (λ_abs_)**: 380–500 nm **Absorption maximum (λ_abs max_)**: ~440 nm **Emission (λ_em_)**: ~500–550 nm (green-yellow fluorescence) **Singlet oxygen quantum yield (Φ_Δ_)**: exact value of the Φ_Δ_ was not provided, but ROS generation was observed	Curcumin-NP-PDT facilitated the maximum usage of curcumin and indicated strong apoptotic effects on tumor cells under LED irradiation

**Table 5:** Past studies on coumarin Ruthenium(II)/Iridium(III) complexes in PDT against cancer.

Topic	Metal Complex & Photophysical Features	Consequence
A near-infrared light-activatable Ru(II)-coumarin photosensitizer active under hypoxic conditions [69]	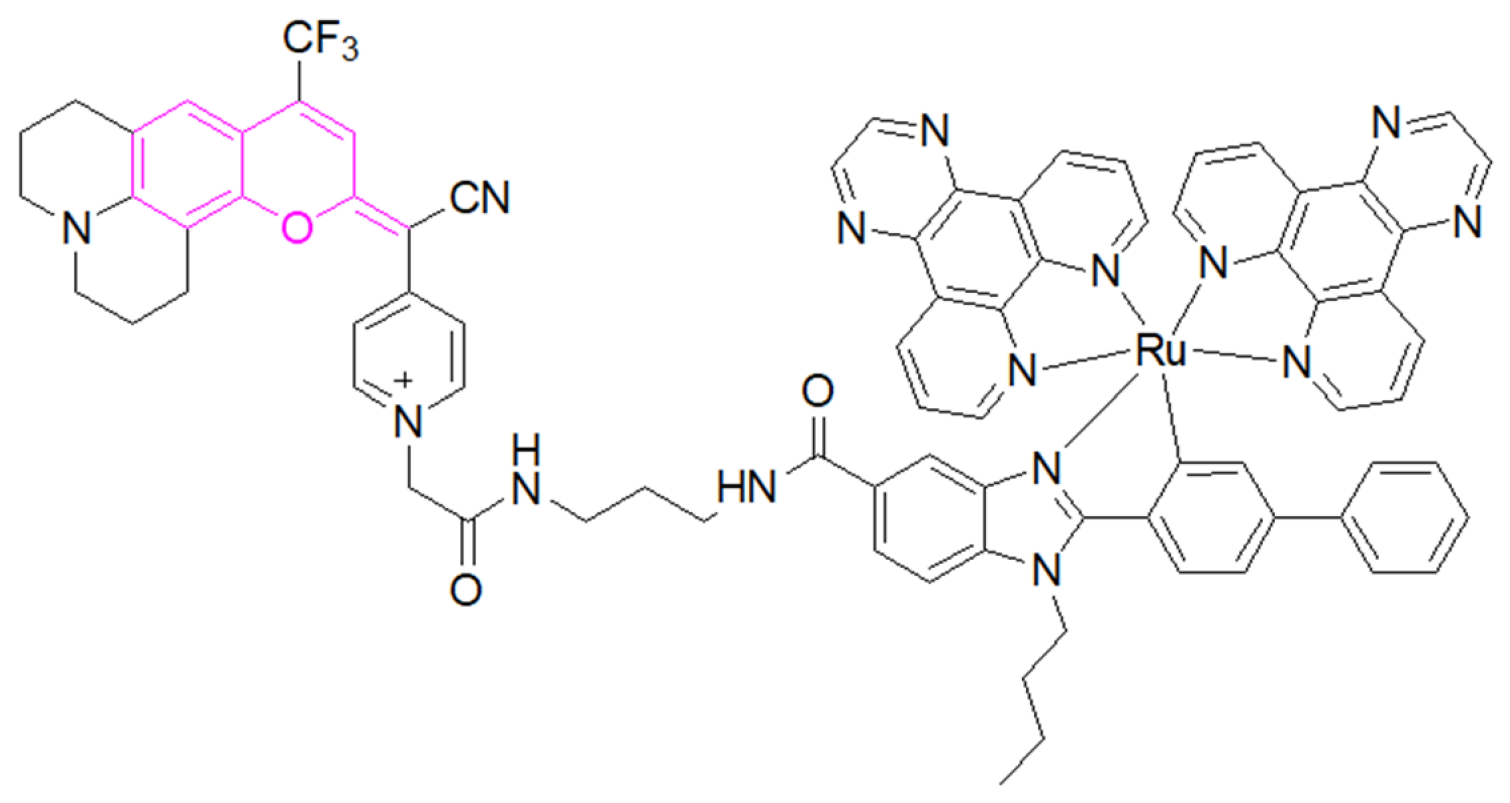 Normoxia (21% O_2_) **IC_50_** (µM, **HT-29**) for Dark: >300, Light: 7.1 ± 0.3 **IC_50_** (µM, **CT-26**) for Dark: >300, Light: 8.6 ± 0.4 **IC_50_** (µM, **HeLa**) for Dark: >300, Light: 6.7 ± 0.5 **IC_50_** (µM, **A2780**) for Dark: >101, Light: 5.9 ± 0.6 Hypoxia (2% O_2_) **IC_50_** (µM, **HT-29**) for Dark: >300, Light: 13 ± 2 **IC_50_** (µM, **CT-26**) for Dark: >300, Light: 15 ± 3 **IC_50_** (µM, **HeLa**) for Dark: >300, Light: 12 ± 2 **IC_50_** (µM, **A2780**) for Dark: >101, Light: 11.8 ± 1.5 **PDT light dose**: 740 nm, 100 mW/cm^2^, 1 h for HT-29; 620 nm, 60 mWcm^−2^, 1 h for CT-26, HeLa, A2780 **Excitation (λ_ex_)**: 532 nm **Absorption (λ_abs_)**: 400–700 nm (broad visible absorption) **Absorption maximum (λ_abs max_)**: ~623 nm **Emission (λ_em_)**: ~600–700 nm (red region phosphorescence) **Singlet oxygen quantum yield (Φ_Δ_)**: ~0.40–0.50	Ru(II)-coumarin conjugate efficiently generated singlet oxygen and superoxide radical anions, thereby achieving high photoactivity toward cancer cells upon highly penetrating 740 nm light irradiation even under hypoxic environments
Ruthenium(II) polypyridyl complexes containing COUBPY ligands as potent photosensitizers for the efficient phototherapy of hypoxic tumors [70]	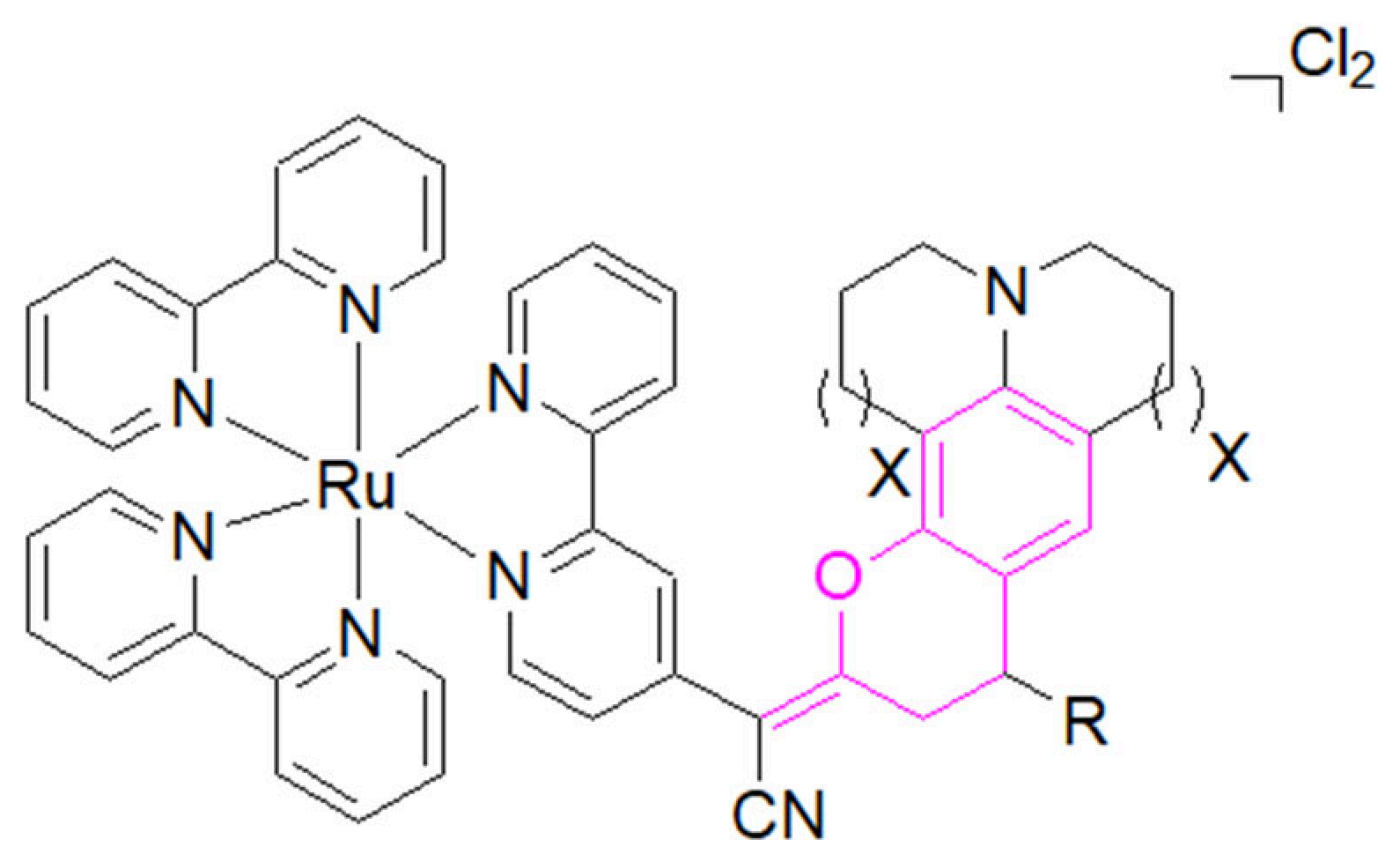 **IC_50_** (µM, **CT-26**) for Dark: >100, Light: ≈ 0.0074 ± 0.0006 at 645 nm under normoxia (21% O_2_) **PDT light dose**: 645 nm, 9 J cm^−2^, 1 h for CT-26 cells; 660 nm, 100 mW/cm^2^, 15 or 20 min for subcutaneous CT-26 tumor model in BALB/c mice **Excitation (λ_ex_)**: 520 nm **Absorption (λ_abs_)**: 400–700 nm (broad visible absorption) **Absorption maximum (λ_abs max_)**: ~520 nm (Q-band region) **Emission (λ_em_)**: 645–670 nm (red region phosphorescence) **Singlet oxygen quantum yield (Φ_Δ_)**: ~0.12–0.21	Ru-COUBPY complex exhibited in vitro cytotoxicity against CT-26 cancer cells when irradiated with light within the phototherapeutic window, achieving nanomolar potency in both normoxic and hypoxic conditions
Coumarin-modified ruthenium complexes: synthesis, characterization, and antiproliferative activity against human cancer cells [71]	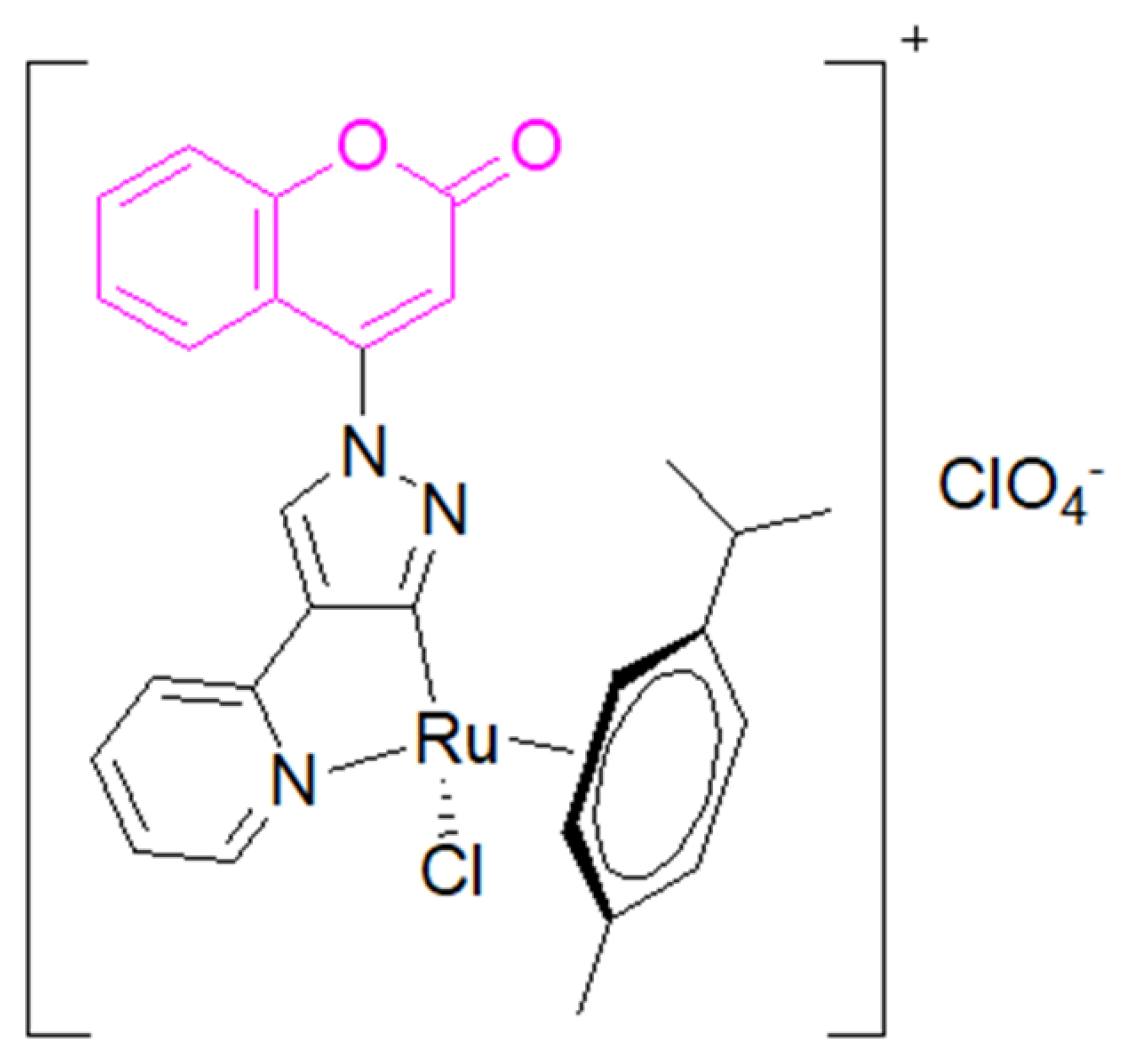 **IC_50_** (µM, **HepG2**) for Dark: >100, Light: 7.8 ± 0.6 **IC_50_** (µM, **PANC-1**) for Dark: >100, Light: 9.4 ± 0.8 **PDT light dose**: ~420–450 nm, 1 h **Excitation (λ_ex_)**: ~420–450 nm (coumarin absorption band) **Absorption (λ_abs_)**: ~420–450 nm (broad visible absorption, dominated by coumarin chromophore) **Absorption maximum (λ_abs max_)**: ~430–450 nm **Emission (λ_em_)**: ~470–500 nm (blue green fluorescence) **Singlet oxygen quantum yield (Φ_Δ_)**: ~0.2–0.3	Coumarin-modified Ru(II) complex regulated the expression and activity of c-Myc and NPM1 in RKO colon carcinoma cells, which induced downregulation of AKT and ERK signaling in PANC-1 cells concomitant with reduced intracellular levels of ROS
Photodynamic effect of light-harvesting, long-lived triplet excited state Ruthenium(II)-polyimine-coumarin complexes: DNA binding, photocleavage and anticancer studies [72]	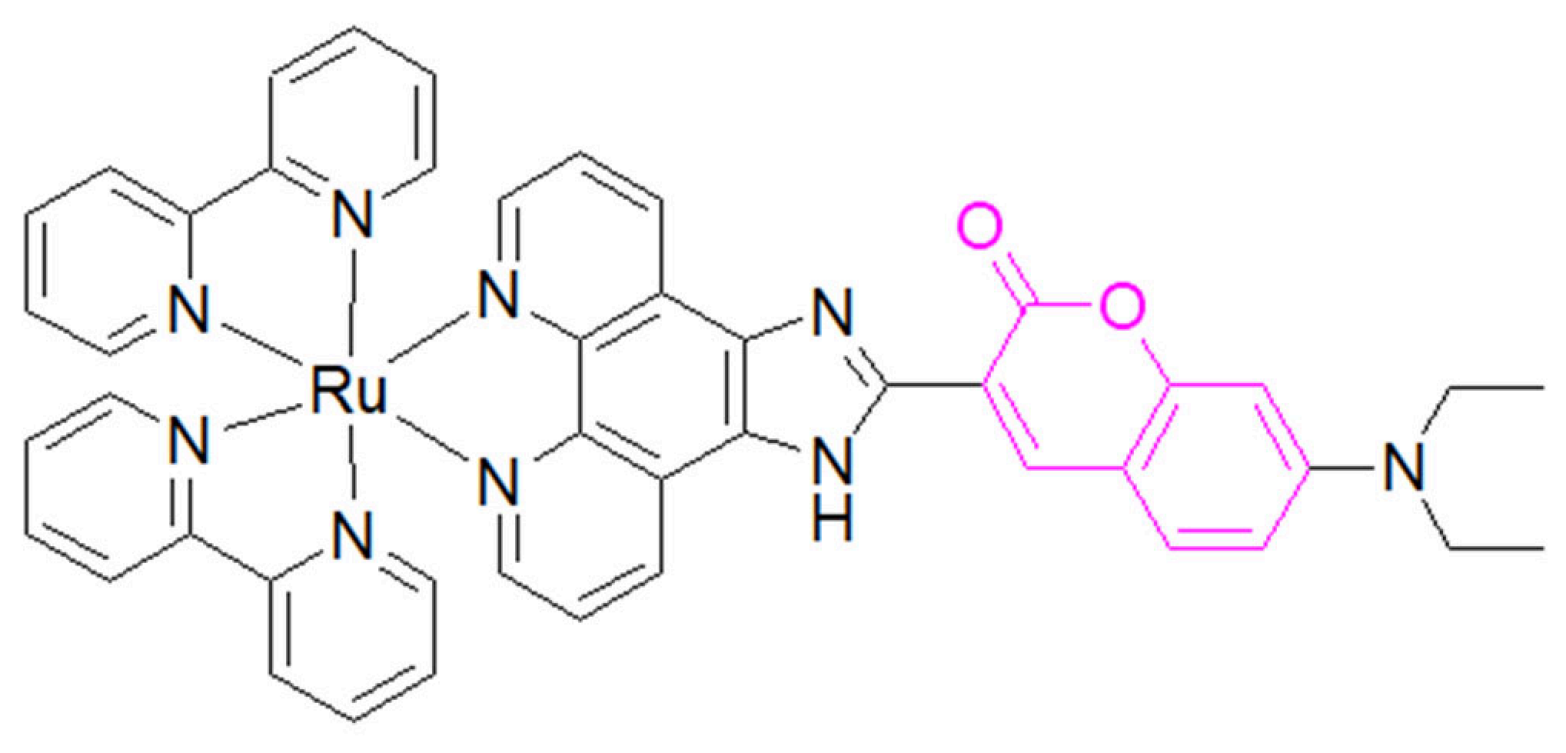 **IC_50_** (µM, **HeLa**) for Dark: >100, Light: 15.5 ± 1.5 **IC_50_** (µM, **BEL-7402**) for Dark: >100, Light: 19.5 ± 1.3 **IC_50_** (µM, **MG-63**) for Dark: >100, Light: 20.0 ± 2.0 **PDT light dose**: 450 nm, 30 min **Excitation (λ_ex_)**: ~490 nm (emission studies); 450 (photo–cleavage assays) **Absorption (λ_abs_)**: ~420–450 nm (broad visible absorption, dominated by coumarin chromophore) **Absorption maximum (λ_abs max_)**: 471 nm **Emission (λ_em_)**: 608 nm (^3^MLCT phosphorescence; fluorescence quenched) **Singlet oxygen quantum yield (Φ_Δ_)**: 0.67	Ru(II)-polyimine-coumarin complex demonstrated efficient cleavage activity, converted complete supercoiled DNA to nicked circular, and shown appreciable cytotoxicity towards the cancer cell lines, such as HeLa, BEL-7402, and MG-63 cells
A cyclometalated Ir(III) complex conjugated to a coumarin derivative is a potent photodynamic agent against prostate differentiated and tumorigenic cancer stem cells [73]	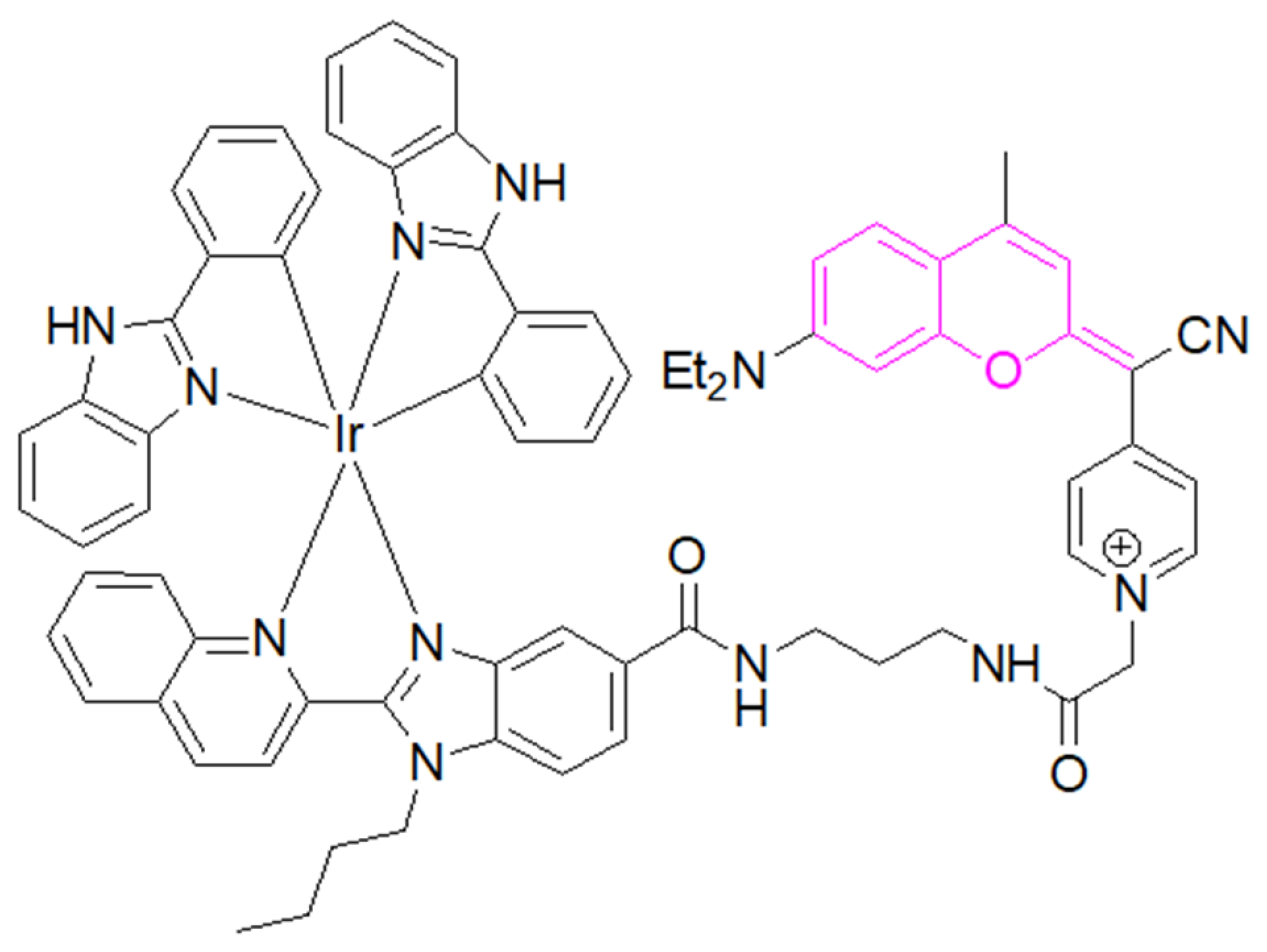 **IC_50_** (µM, **DU145**) for Dark: >100, Light: ~2–5 µM (exact EC_50_ from survival curves) **PDT light dose**: 420 nm, 28 J cm^−2^, 30 min **Excitation (λ_ex_)**: ~420 nm (coumarin absorption band) **Absorption (λ_abs_)**: ~420–450 nm (broad visible absorption, dominated by coumarin chromophore) **Absorption maximum (λ_abs max_)**: 420 nm **Emission (λ_em_)**: ~510 nm (green fluorescence suitable for Ca^2+^ flux assays) **Singlet oxygen quantum yield (Φ_Δ_)**: ~0.6–0.7	A cyclometalated Ir(III) complex conjugated to a coumarin derivative induced apoptotic cell death in prostate cancer DU145 cells associated with calcium signaling flux in these cells and autophagy
Exploring structure-activity relationships in photodynamic therapy anticancer agents based on Ir(III)-COUPY conjugates [74]	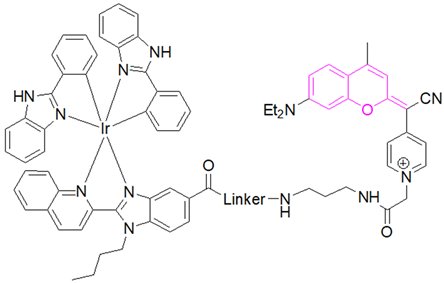 Normoxia (21% O_2_) **IC_50_** (µM, **A375**) for Dark: >100, Light: 18 ± 3 **IC_50_** (µM, **SK-MEL-28**) for Dark: >100, Light: 1.5 ± 0.1 **IC_50_** (µM, **HeLa**) for Dark: >100, Light: 8.6 ± 0.7 **IC_50_** (µM, **A2780**) for Dark: >100, Light: 1.07 ± 0.07 **IC_50_** (µM, **A2780 cis**) for Dark: >100, Light: 0.70± 0.06 **PDT light dose**: 520 nm, 1.5 mW cm^−2^, 1 h **Excitation (λ_ex_)**: 520 nm (MLCT band excitation) **Absorption (λ_abs_)**: ~400–600 nm (broad visible absorption) **Absorption maximum (λ_abs max_)**: ~510–520 nm **Emission (λ_em_)**: ~600–620 nm (^3^MLCT phosphorescence) **Singlet oxygen quantum yield (Φ_Δ_)**: ~0.81	Ir(III)-COUPY conjugates exhibited high phototoxicity under green light irradiation, which was attributed to the photogeneration of ROS, and remained non-toxic in the dark
Highly efficient Ir(III)-coumarin photo-redox catalyst for synergetic multi-mode cancer photo-therapy [75]	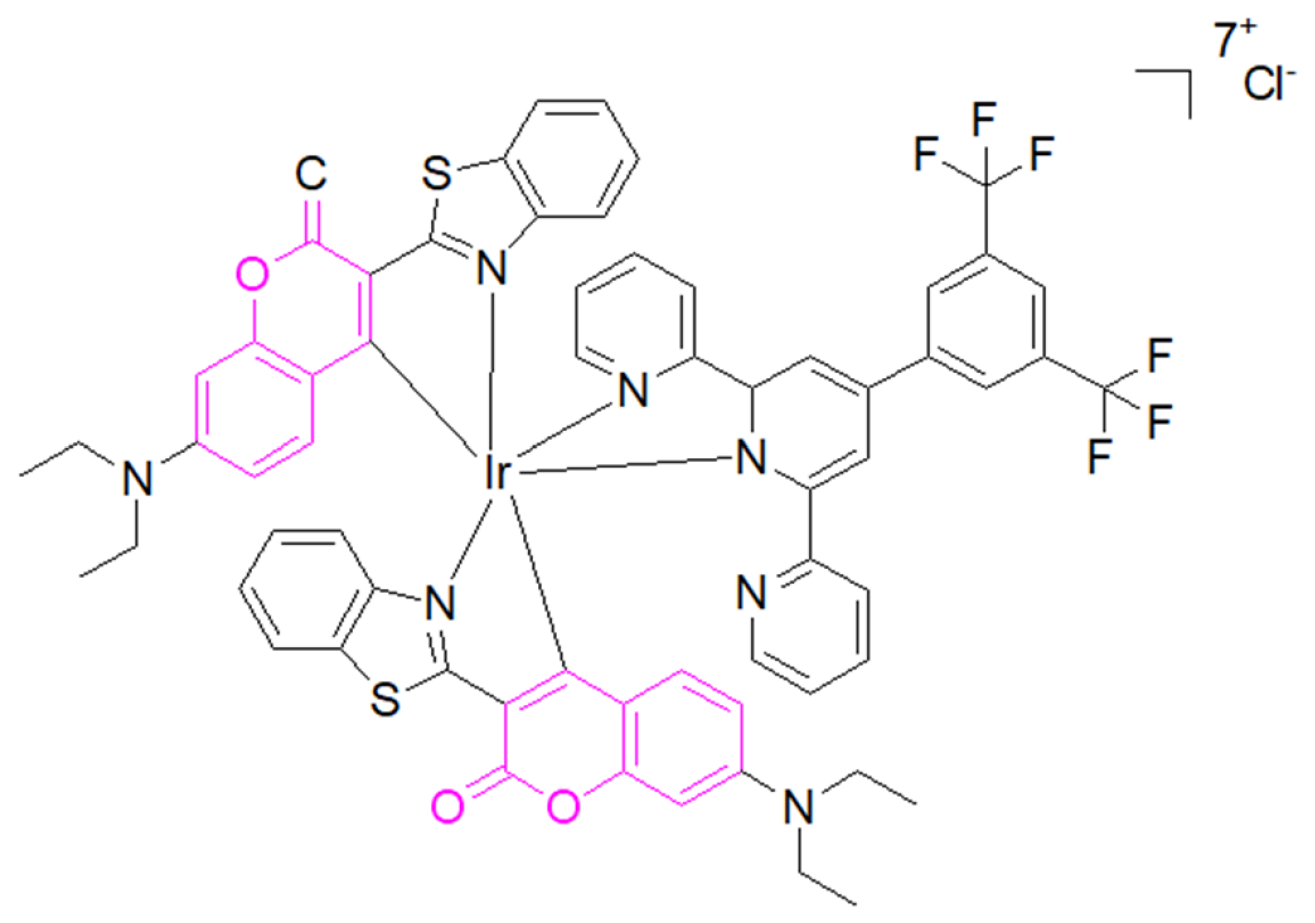 **IC_50_** (µM, **HeLa**) for Dark: 23.8, Light: 0.03 (465 nm, 11.7 J/cm^2^), 0.1 (525 nm, 29.56 J/cm^2^) **IC_50_** (µM, **B16**) for Dark: 37.7, Light: 1.1 (465 nm, 11.7 J/cm^2^) **IC_50_** (µM, **A431**) for Dark: 3.8, Light: 0.01 (465 nm, 11.7 J/cm^2^), 0.01 (525 nm, 29.56 J/cm^2^) **IC_50_** (µM, **NP69**) for Dark: 11.9, Light: 0.5 (465 nm, 11.7 J/cm^2^) **PDT light dose**: 465 nm, 11.7 J/cm^2^; 525 nm, 29.56 J/cm^2^ **Excitation (λ_ex_)**: ~465 nm and 525 nm (coumarin absorption region) **Absorption (λ_abs_)**: ~400–600 nm (broad visible absorption) **Absorption maximum (λ_abs max_)**: ~470–480 nm **Emission (λ_em_)**: ~607 nm **Singlet oxygen quantum yield (Φ_Δ_)**: ~0.65–0.70	Ir(III)-coumarin complex triggered photo-induced intracellular redox imbalance by NAD(P)H oxidation and ROS generation, as well as changes in the mitochondrial membrane potential
Novel heteroleptic Iridium(III) complexes containing COUBPY ligands for effective photoinduction of ferroptosis for cancer therapy [76]	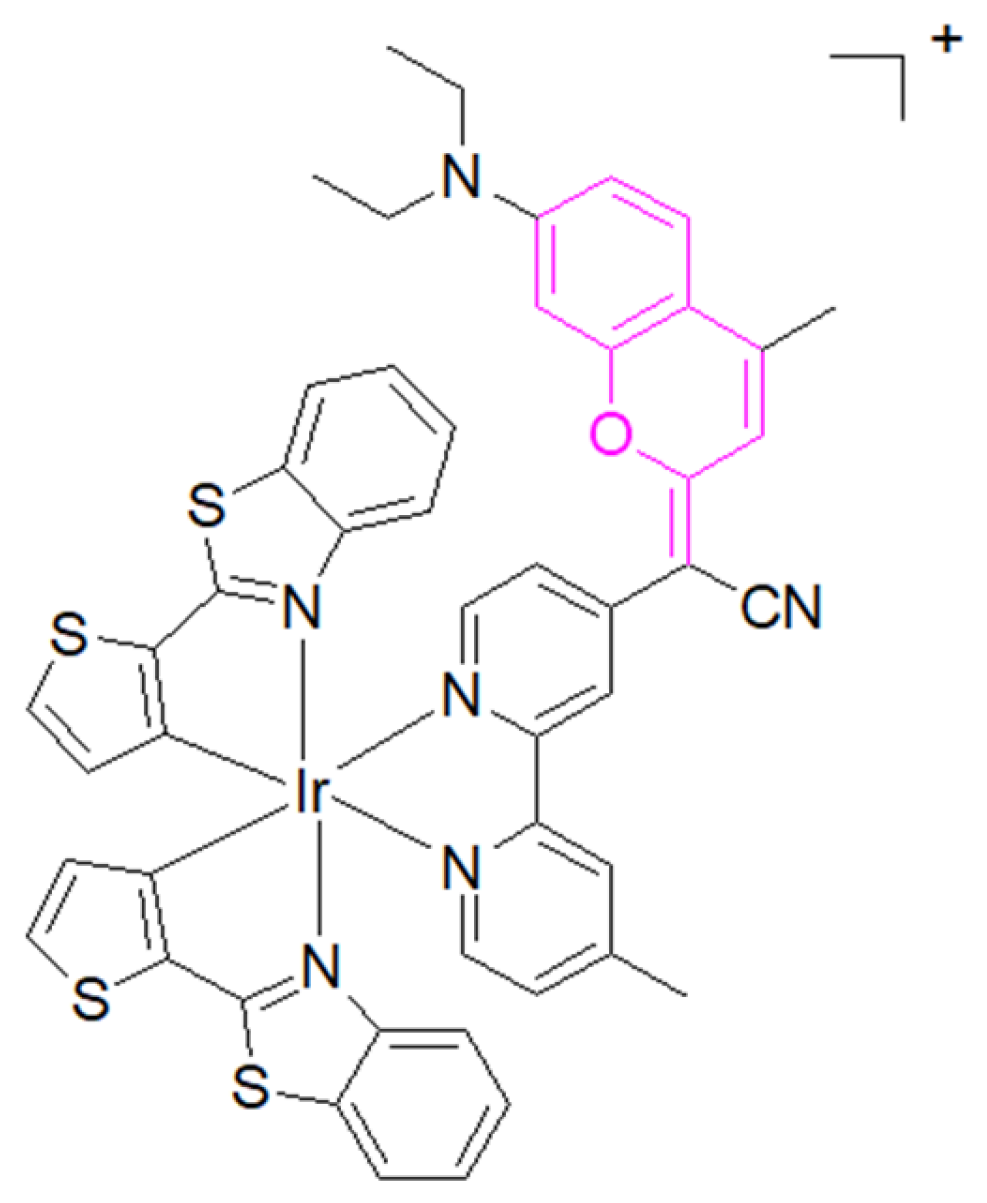 **IC_50_** (µM, **A375**) for Dark 17.6 ± 4.7, Light: 0.098 ± 0.025 (520 nm, 1.5 mW/cm^2^), 0.290 ± 0.031 (620 nm, 15 mW/cm^2^) **IC_50_** (µM, **HeLa**) for Dark 17.3 ± 4.2, Light: 0.112 ± 0.018 (520 nm, 1.5 mW/cm^2^), 0.687 ± 0.118 (620 nm, 15 mW/cm^2^) **PDT light dose**: 520 nm, 1.5 mW/cm^2^ or 620 nm, 15 mW/cm^2^ **Excitation (λ_ex_)**: 520 nm (green light, 2.0 mW cm^−2^) and 620 nm (red light, 15 mW cm^−2^) **Absorption (λ_abs_)**: ~300–700 nm (broad visible absorption) **Absorption maximum (λ_abs max_)**: ~520 nm (green band), ~580 nm (red band) **Emission (λ_em_)**: 600–700 nm (red phosphorescence, suitable for imaging) **Singlet oxygen quantum yield (Φ_Δ_)**: 0.57	Coumarin-based COUBPY ligand Ir(III) complex exhibited high stability under both dark and light conditions, which photogenerated Type I and Type II ROS, as well as photo-oxidizing NADH
Anticancer profile of coumarin 6-based Ir(III) photocatalysts under normoxia and hypoxia by ROS generation and NADH oxidation [77]	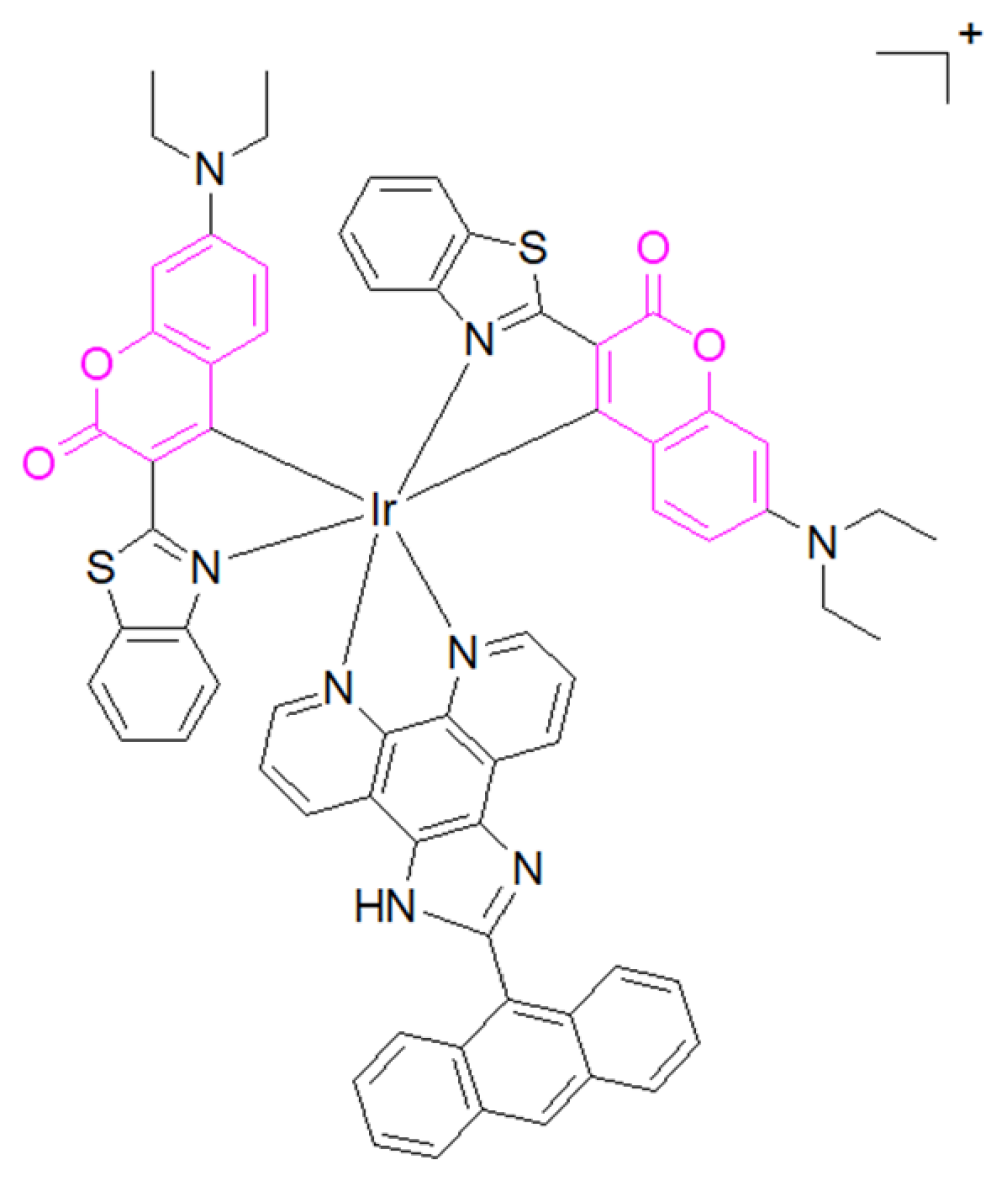 **IC_50_** (µM, **A549**) for Dark 42.1 ± 2.7, Light: 0.7 ± 0.1 **IC_50_** (µM, **HeLa**) for Dark ≈42 ± 2, Light: ≈0.8 ± 0.1 **PDT light dose**: 400–700 nm, 1 h (10 J cm^−2^) **Excitation (λ_ex_)**: 525 nm (NADH oxidation assays) **Absorption (λ_abs_)**: 250–700 nm **Absorption maximum (λ_abs max_)**: 346, 365, 385, 456, 478 nm **Emission (λ_em_)**: ~495 nm (green fluorescence) **Singlet oxygen quantum yield (Φ_Δ_)**: exact value of the Φ_Δ_ was not provided, but ROS generation was observed	Coumarin 6-based Ir(III) photocatalysts demonstrated photo-toxicity, which was localized in the mitochondria, generated ROS, and oxidized NADH during light exposure, ultimately causing cell apoptosis via mitochondrial depolarization

**Table 6:** Past studies on curcumin Ru(II)/Ir(III) complexes in PDT against cancer.

Topic	Metal Complex & Photophysical Features	Consequence
Ruthenium(II)-arene curcuminoid complexes as photosensitizer agents for antineoplastic and antimicrobial photodynamic therapy: *in vitro* and *in vivo* insights [78]	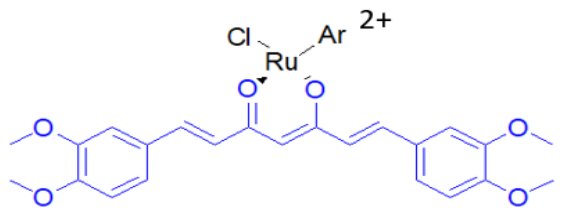 **IC_50_** (µM, **HCT116**) for Dark: not numerically specified, Light: 1.06 ± 0.23 **IC_50_** (µM, **HT-29**) for Dark: not numerically specified, Light: 1.59 ± 0.27 **PDT light dose**: 400–700 nm (halogen white lamp), 30 min **Excitation (λ_ex_)**: 450–650 nm (broad visible excitation under halogen lamp) **Absorption (λ_abs_)**: 300–600 nm (broad visible absorption) **Absorption maximum (λ_abs max_)**: ~420–430 nm (curcumin absorption region) Emission (λ_em_): ~500–550 nm (green fluorescence) **Singlet oxygen quantum yield (Φ_Δ_)**: ~0.2–0.3	Ru(II)-arene curcuminoid complex improved curcumin activity to induce ROS production, apoptosis, necrosis, and autophagic cell death in colon cancer cells
Two in one: merging photoactivated chemotherapy and photodynamic therapy to fight cancer [79]	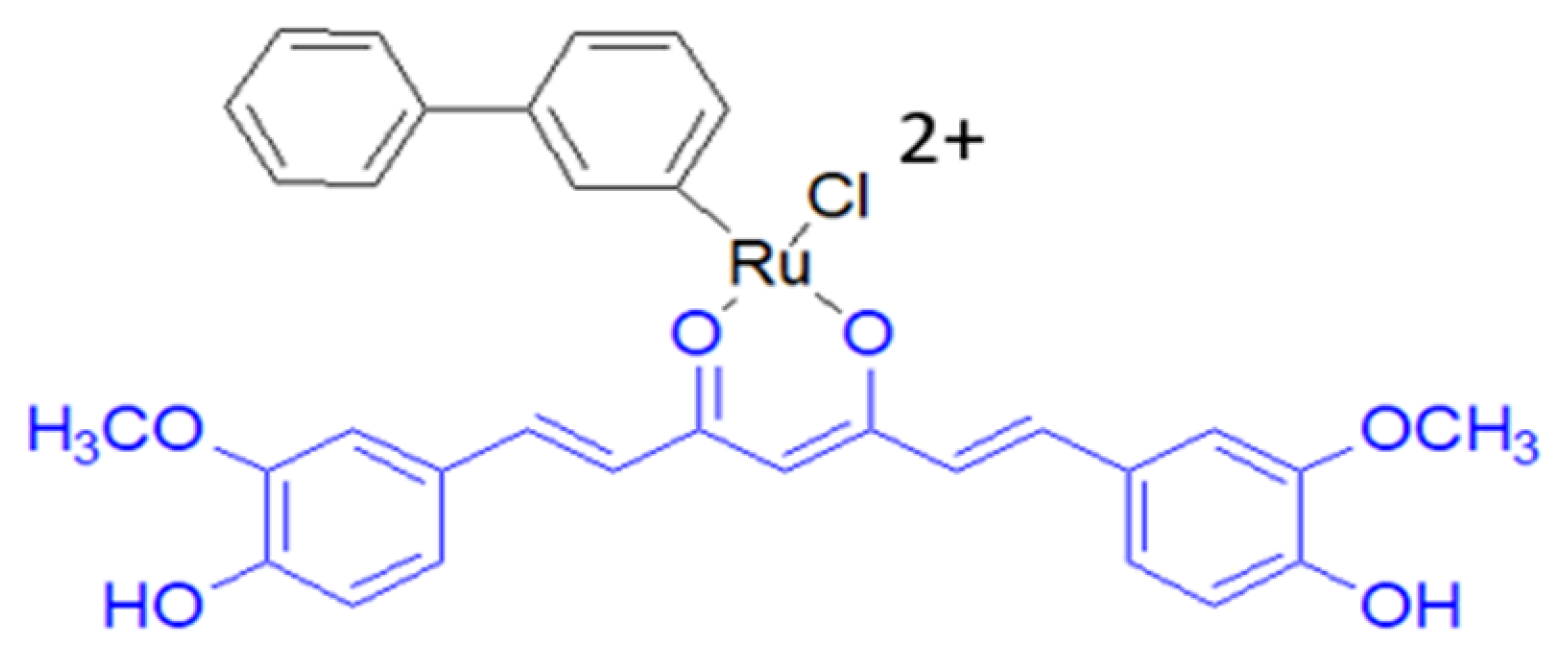 **IC_50_** (µM, **HeLa**) for Dark: ~4.8, Light: ~0.26 **IC_50_** (µM, **A549**) for Dark: ~5–6, Light: ~0.3–0.4 **IC_50_** (µM, **MCF-7**) for Dark: ~5, Light: ~0.35 **PDT light dose**: 420–450 nm (blue light), 10–20 J/cm^2^ **Excitation (λ_ex_)**: 420–450 nm (blue light) **Absorption (λ_abs_)**: 400–500 nm (broad visible absorption) **Absorption maximum (λ_abs max_)**: 473 nm, 531 nm **Emission (λ_em_)**: 600–650 nm **Singlet oxygen quantum yield (Φ_Δ_)**: exact numerical value not specified, but reported as moderate to high	Ru(II)-curcumin complex exemplified a promising “two-in-one” agent that combined efficient ROS production with ligand release to overcome tumor hypoxia and enhanced therapeutic selectivity
A highly luminescent tetrahydrocurcumin Ir(III) complex with remarkable photoactivated anticancer activity [80]	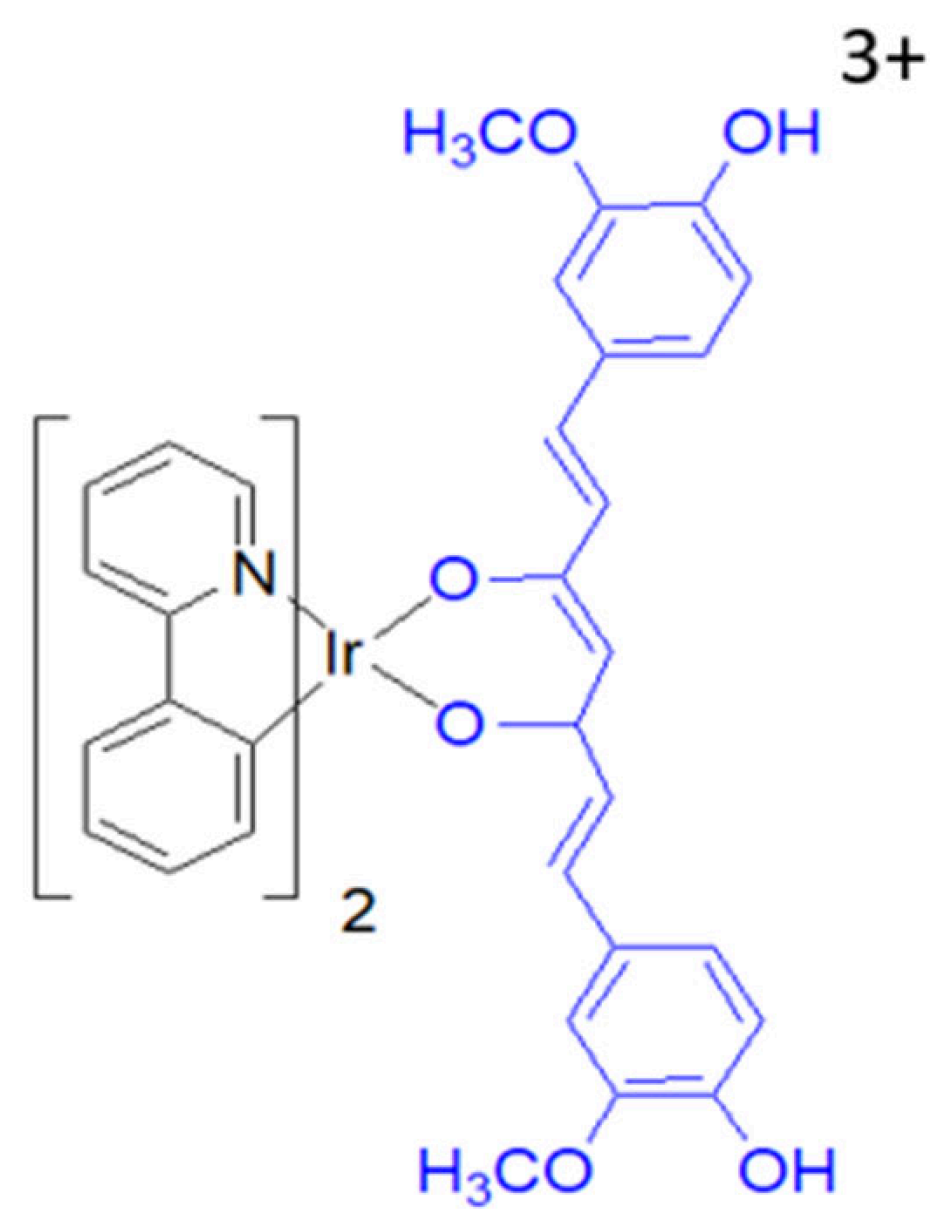 **IC_50_** (µM, **HeLa**) for Dark: not numerically specified, Light: ~5.2 **IC_50_** (µM, **A549**) for Dark: not numerically specified, Light: ~6.4 **PDT light dose**: 465–495 nm, ~10–20 J/cm^2^, 2–20 s **Excitation (λ_ex_)**: 465–495 nm (blue light) **Absorption (λ_abs_)**: 400–500 nm (broad visible absorption) **Absorption maximum (λ_abs max_)**: ~520 nm **Emission (λ_em_)**: ~600 nm **Singlet oxygen quantum yield (Φ_Δ_)**: 0.98	Ir(III) THC complex was highly luminescent in deoxygenated solution and efficiently generated singlet oxygen under aerated conditions, inducing rapid apoptosis for PDT

**Table 7:** Comparison of Ru(II) and Ir(III) complexes in PDT immunotherapy.

Feature	Ruthenium(II) Complexes	Iridium(III) Complexes
Photophysical properties	Strong absorption in visible/red light; Efficient ^1^O_2_ and ROS generation; Good cellular uptake	Long-lived triplet excited states; Highly efficient ROS generation; Mitochondrial localization
Mechanism of ICD induction	ROS → ER stress → DAMP release (calreticulin, ATP, HMGB1) → dendritic cell activation	ROS → oxidative stress → DAMP release (calreticulin, ATP, HMGB1)→ dendritic cell activation
Immune effects	Prime of CD8^+^ cytotoxic T-cells; Systemic antitumor immunity; Synergy with checkpoint blockade	Enhance CD8^+^ infiltration; Synergy with checkpoint blockade; Conversion of “cold” tumors into “hot”
Cancer models studied	HeLa cells (cervical cancer), colorectal cancer (*in vivo*)	Multiple solid tumors; Mitochondrial-targeted Ir(III) complexes in digestive cancers
Selectivity	Strong phototoxicity under light, inert in dark → therapeutic safety	Potent PDT efficacy, low dark toxicity → therapeutic selectivity
Advantages	Demonstrate systemic immunity in colorectal cancer; Checkpoint blockade synergy	Mitochondrial targeting, Ferritinophagy-mediated ICD; Ferroptosis-like mechanisms amplify immune response

**Table 8:** Comparison of conventional and stimuli-responsive nanocarriers.

Type	Nanocarrier	Mechanism	Advantages	Limitations	References
Conventional	Liposomes; polymeric Nanoparticles; Dendrimers; Cubosomes	Passive tumor targeting via EPR effect; Stabilization of hydrophobic Ru/Ir complexes; Control release	Improve solubility; Prolong circulation; Protect complexes from degradation	Limit selectivity; Risk of off-target accumulation	[32,40, 41,45]
Stimuli- Responsive	pH-sensitive, ROS- responsive; Enzyme- triggered systems	Release triggered by tumor microenvironment cues (acidic pH, oxidative stress, protease activity)	High selectivity; Reduce systemic toxicity; Enhance therapeutic efficacy	Complex design; Tumor heterogeneity	[47]
Hybrid Nanoplatforms	Upconversion nanoparticles; X-ray scintillator-based systems	Extend activation wavelengths into NIR	Overcome tissue penetration; Deep-seated tumor treatment	Require specialized light sources or radiation	[45]

**Table 9:** Comparison of clinically approved PSs for PDT.

Feature	Photofrin^®^ (Porfimer Sodium)	Verteporfin (Visudyne^®^)	Talaporfin Sodium (NPe6)
Type	First-generation	Second-generation	Second-generation
Chemical structure	Hematoporphyrin derivative	Benzoporphyrin derivative	Chlorin derivative
Excitation wavelength (λ_ex_)	~630 nm	~689 nm	~664 nm
Tissue penetration	5–10 mm (shallow)	Deeper than photofrin	Deeper than photofrin
Drug clearance	Slow, weeks	Faster, days	Faster, days
Skin photosensitivity	Prolong	Shorter duration	Shorter duration
Tumor selectivity	Limit	Liposomal, LDL receptor targeting	Improve selectivity via chlorin structure
Clinical approval	Esophageal cancer, NSCLC, Barrett’s dysplasia	Age-related macular degeneration, Oncology trials	Lung cancer and other solid tumors approved in Japan
Limitations	Shallow penetration, Prolong, Photosensitivity, Oxygen dependence	Oxygen-dependent	Oxygen dependence remains, but better photophysical yield

**Table 10:** Reasons for using natural ligands (coumarin and curcumin) and metal ions (Ru(II)/Ir(III)).

	Features	Advantages	Limitations
Coumarin (Natural Ligand)	Benzopyrone scaffold; Blue fluorescence; Low Φ_Δ_ < 0.1 [23]	Antioxidant, anti-inflammatory, anticancer; Biocompatible to reduce heavy-metal toxicity [13,14,15,16,17]	Low ROS generation; Require metal ion coordination to enhance PDT efficiency
Curcumin (Natural Ligand)	Polyphenolic β-diketone; Broad emission; Φ_Δ_ from ~0.2–0.6 [24]	Antioxidant, anti-inflammatory, anticancer; Improve solubility and bioavailability; Synergize with metal ions [18,19,20,21,22]	Moderate ROS yield; Photostability problem, e.g., degradation
Ruthenium(II) Metal Ion	Strong spin-orbit coupling; Broad visible absorption; Red phosphorescence [25,26,27,28,29,30]	Efficient ISC; High ROS yield; Hypoxia tolerance; Multimodal activity in apoptosis, ferroptosis, immune activation [32,33,34,35,36,37,38,39,40,41,42]	Heavy-metal toxicity risk; Require delivery systems
Iridium(III) Metal Ion	Strong red/NIR luminescence; Φ_Δ_ up to ~0.78; Organelle-specific targeting [25,26,27,28,29,30,31]	NIR deep tissue penetration; Dual theranostics for therapy and imaging; Highly effective under hypoxia [43,44,45,46,47]	Rare; Expensive; Long-term accumulation

**Table 11:** Comparative photophysical properties of free ligands and Ru(II)/Ir(III) complexes.

Photophysical Feature	Coumarin (Ligand) [23]	Curcumin (Ligand) [24]	Ru(II)/Ir(III)- Coumarin Complexes [32,40]	Ru(II)/Ir(III)- Curcumin Complexes [36,44,45]
Absorption range	UV–blue (320–380 nm)	Visible (410–430 nm)	Extended into red or NIR (500–650 nm)	Extended into visible or NIR (500–700 nm)
Singlet oxygen quantum yield (Φ_Δ_)	Very low (<0.1)	Moderate (~0.2–0.3)	High (~0.67)	High (~0.6–0.78)
ISC efficiency	Limited	Moderate	Strong via spin-orbit coupling	Strong via spin-orbit coupling
Photostability	Moderate, prone to degradation	Poor solubility, unstable	Improve stability	Enhance stability with nanocarriers
Photocytotoxicity	Micromolar IC_50_ values	Micromolar IC_50_ values	Low micromolar to nanomolar IC_50_ values	Nanomolar IC_50_ values, even under hypoxia

**Table 12:** Comparison of conventional organic PSs (e.g., porphyrins, BODIPY) with coumarin and curcumin.

Feature	Porphyrins	BODIPY	Coumarin	Curcumin
Chemical structure	Tetrapyrrolic macrocycle 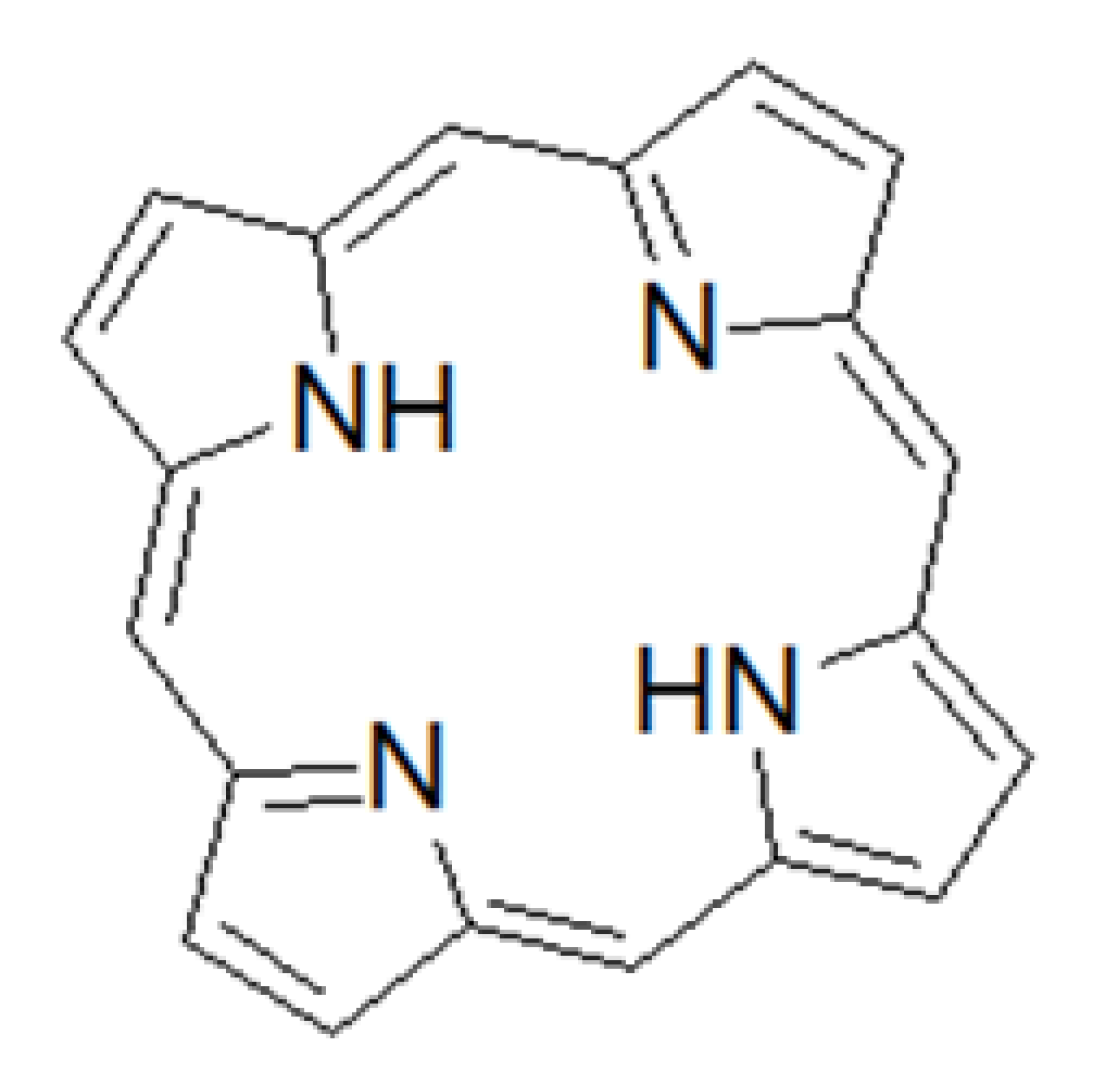	Boron-dipyrromethene core 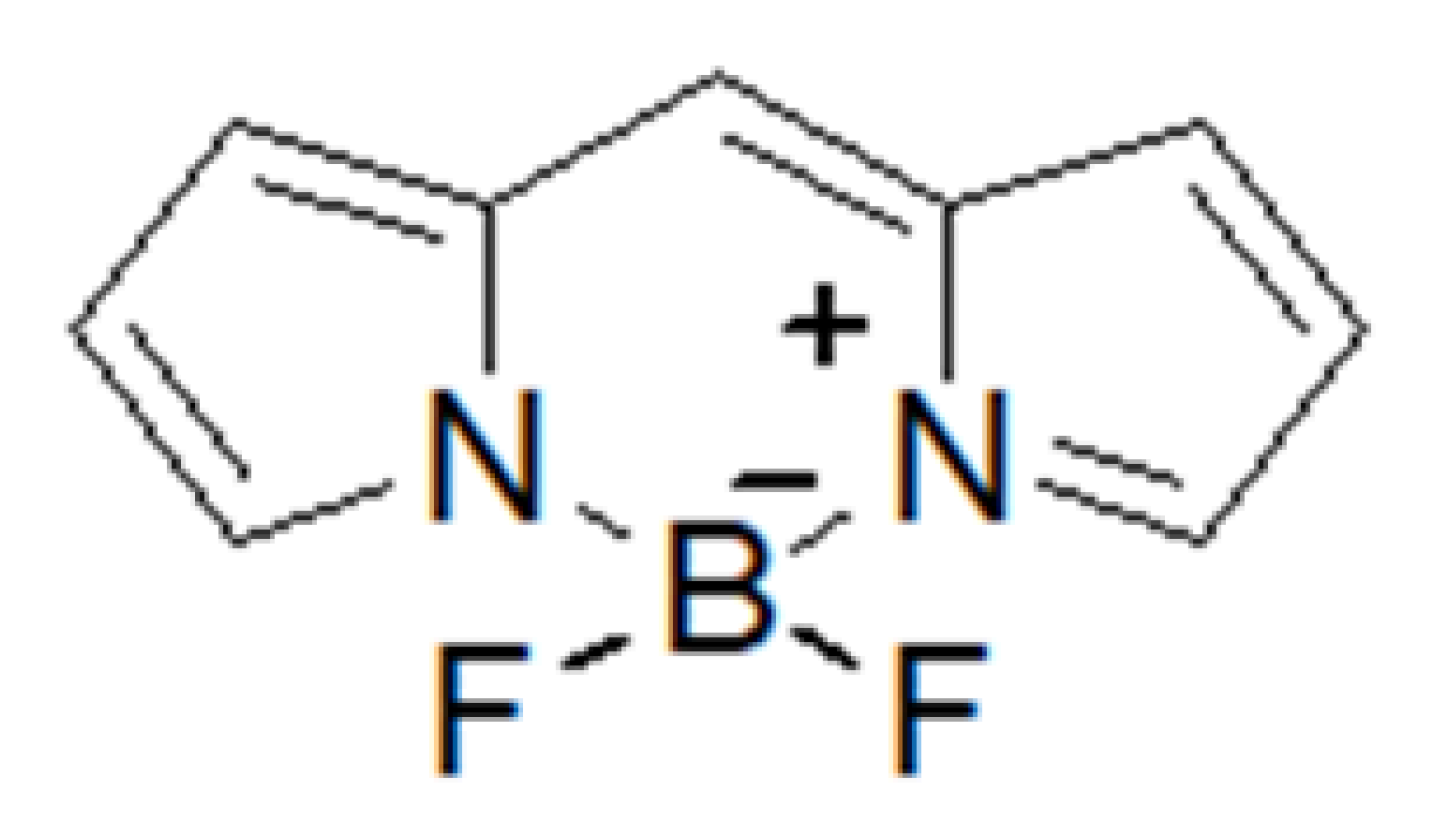	Benzopyrone scaffold 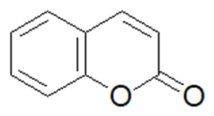	Polyphenolic β-diketone 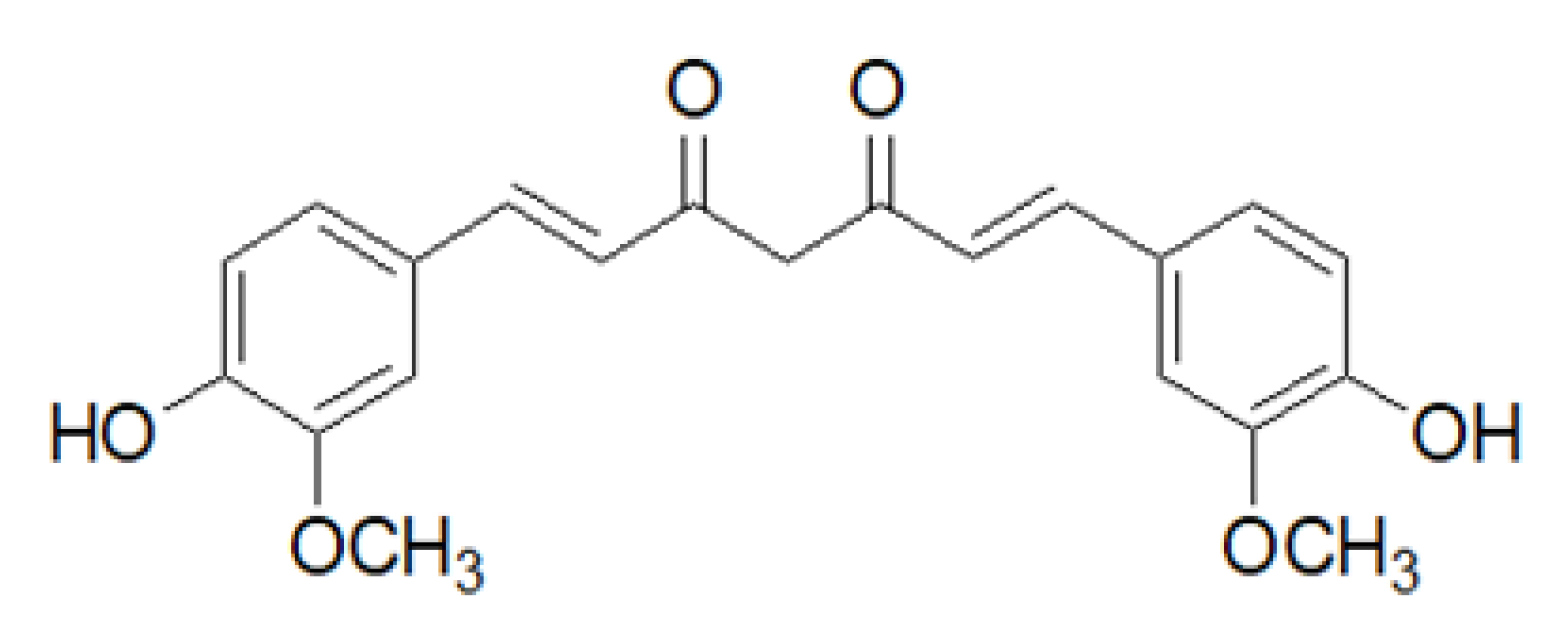
Structural- activity relationship	π-extension and donor-acceptor substitution improve λ_abs_ and solubility; Supramolecular assembly to enhance tumor targeting [105]	Substituent modification by PEGylation, glycosylation, halogenation to tune λ_abs_/λ_em_; Photostability; Organelle targeting [106]	Electron- donating or -withdrawing groups for fluorescence; ROS yield; Limit SAR for PDT with low Φ_Δ_ < 0.1 [107]	Influence ROS yield; nanoformulations improve stability and bioavailability [108]
Absorption (λ_abs_)	400–700 nm (broad visible/NIR); Q-bands; Red region [109]	~500–600 nm (visible/NIR) [110]	~320–380 nm (peak ~350 nm) [23]	~410–430 nm [24]
Emission (λ_em_)	~650–700 nm (red/NIR); Strong fluorescence [109]	500 nm to 800 nm (red/NIR); Strong fluorescence [110]	~400–480 nm (blue fluorescence) [23]	~460–550 nm [24]
Photostability	Stable macrocycles; Porphyrin-polymer conjugates reduce photobleaching [111]	Resistant to photobleaching; Heavy-atom-free improve stability [112]	Susceptible to photobleaching [113,114]	Moderate; Improve with nanocarriers [108]
Clinical status	Clinically approved (e.g., photofrin) for fluorescence-guided PDT [104]	Preclinical or experimental for multifunctional imaging or PDT agents [105]	Preclinical study only [109]	Preclinical study only [115,116]

**Table 13:** Comparison of coumarin and curcumin in PDT mechanism.

Feature	Coumarin	Curcumin
Absorption (λ_abs_)	~320–480 nm (blue fluorescence)	~410–550 nm (broad visible)
Singlet oxygen quantum yield (Φ_Δ_)	<0.1 (Very low)	~0.2–0.6 (Moderate)
Favored PDT mechanism	Type I (radical-mediated, O_2_-independent)	Type II (^1^O_2_ generation, O_2_-dependent)
Strengths	Hypoxic tumors; radical generation	Higher Φ_Δ_; effective in normoxia
Limitations	Weak ROS yield; poor photostability	Moderate stability; limited penetration
Applications	Coordinate with Ru(II)/Ir(III) complexes for dual Type I/II PDT	PS for conventional PDT; stronger clinical potential

## Data Availability

No new data were created or analyzed in this study. Data sharing is not applicable to this article.

## Supplementary Materials

The following supporting information can be downloaded at: https://www.mdpi.com/article/10.3390/ijms27177585/s1.

## Abbreviations

The following abbreviations are used in this manuscript:

APCs	Antigen-Presenting Cells
AI	Artificial Intelligence
BODIPY	Boron-Dipyrromethene
CAR-T	Chimeric Antigen Receptor T cell
CD8^+^	T-cell Cluster of Differentiation 8 Positive T-cell
CNKI	China National Knowledge Infrastructure
COX-2	Cyclooxygenase-2
DNA	Deoxyribonucleic Acid
DPPH	2,2-Diphenyl-1-picrylhydrazyl
EMA	European Medicines Agency
ER	Endoplasmic Reticulum
FDA	Food and Drug Administration
IC_50_	Half Maximal Inhibitory Concentration
ICD	Immunogenic Cell Death
IC	Internal Conversion
ISC	Intersystem Crossing
iNOS	Inducible Nitric Oxide Synthase
Ir(III)	Iridium(III)
JAK/STAT	Janus Kinase/Signal Transducer and Activator of Transcription
ML	Machine Learning
MAPK	Mitogen-Activated Protein Kinase
NF-κB	Nuclear Factor kappa-light-chain-enhancer of activated B cells
NIR	Near-Infrared
NMPA	National Medical Products Administration (China)
NO	Nitric Oxide
O_2_^•−^	Superoxide Radical
PK	Pharmacokinetics
PDT	Photodynamic Therapy
PEG-PLGA	Polyethylene Glycol-Poly(lactic-co-glycolic acid)
PGE_2_	Prostaglandin E_2_
PS	Photosensitizer
Pt(IV)	Platinum(IV)
Q-band	Quasi-band (absorption region in porphyrins)
ROS	Reactive Oxygen Species
Ru(II)	Ruthenium(II)
S0, S1, S2	Singlet Ground and Excited States
SVCT2	Sodium-Dependent Vitamin C Transporter 2
T1	Triplet Excited State
TCM	Traditional Chinese Medicine
TNF-α	Tumor Necrosis Factor Alpha
Φ_Δ_	Singlet Oxygen Quantum Yield
