# Supplementary material for: Coumarin and Curcumin-Metal Complexes as Next-Generation Photosensitizers in Cancer Photodynamic Therapy
Source: Int J Mol Sci. 2026 Aug 24;27(17):7585. doi: 10.3390/ijms27177585 (PMC13566110; doi:10.3390/ijms27177585)
Supplement: Supplementary file 1 [file ijms-27-07585-s001.zip › ijms-4499665-supplementary.pdf]

File S1: **Narrative Review Checklist**

Section/Topic	Item No	Item	Reported on Section/Paragraph
**TITLE**			
Title	1	Identify the report as a Narrative Review or Literature Review.	Title: "Coumarin and Curcumin–Metal Complexes as Next-Generation Photosensitizers in Cancer Photodynamic Therapy", explicitly identified as a Review.
**ABSTRACT**			
Structured summary	2	Provide a structured summary with the subsections as: objective, background, methods, conclusion.	Abstract: Structured with Objectives, Methods, Results, Conclusions; includes immune effects, translational, and clinical approval considerations.
**INTRODUCTION**			
Rationale/background	3	Describe the rationale for the review in the context of what is already known.	Introduction: Explains rationale for metal–ligand complexes in PDT, advantages (heavy atom effect, NIR penetration, oxygen independence), drawbacks (toxicity, off-target effects), and role of natural ligands.
Objectives	4	Specify the key question(s) identified for the review topic.	Key questions — role and properties of natural ligands (coumarin, curcumin), coordination with Ru/Ir complexes, photophysical and immunological mechanisms, translational potential in PDT oncology.
**METHODS**			
Research selection	5	Specify the process for identifying the literature search (eg, years considered, language, publication status, study design, and databases of coverage).	Search Strategy: Publications from 2010–2025, databases (WanFang Data, PubMed, ScienceDirect, Scopus, Web of Science, Springer Link, SciFinder, CNKI), no language restrictions, inclusion/exclusion criteria, 35 studies selected.
**DISCUSSION/SUMMARY**			
Narrative	6	Discuss: 1) research reviewed including fundamental or key findings, 2) limitations and/or quality of research reviewed, and 3) need for future research.	Results & Discussion: Findings — natural ligands have intrinsic bioactivity but limited photophysics; Ru/Ir complexes extend absorption, improve ROS yield, induce ICD, and synergize with checkpoint blockade. Limitations — heavy-metal toxicity, delivery challenges. Future research — ligand engineering, nanotechnology integration, translational models.
Summary	7	Provide an overall interpretation of the narrative review in the context of clinical practice for health professionals, policy development and implementation, or future research.	Conclusions: Natural ligand–metal complexes are promising next-generation PDT agents; integration of natural bioactivity with advanced photophysical properties offers translational potential. Clinical approval depends on safety, pharmacokinetics, and nanodelivery validation. Future directions emphasize bridging preclinical promise with clinical translation.

Table S1. 35 selected studies on coumarin/curcumin-derived Ru(II) and Ir(III) complexes in cancer PDT.

Study No.	Reference	Metal Complex	Natural Ligand	Cancer Model (s)	Mechanism (s)	Outcome
1	[32]	Ru(II) polypyridyl	Coumarin	A549, 4T1	ROS, ICD	$CD8^+$ T cell activation, tumor inhibition 94–97%
2	[33]	Ru(II) porphyrin	Coumarin	AGS gastric	ER stress, apoptosis	Caspase-3 mediated apoptosis
3	[34]	Ru(II) radioPDT	Curcumin	PC3 prostate	ROS via X-ray	Comparable to PPIX radioPDT
4	[35]	Ru(II) bipyridine	Coumarin	HeLa, CT-26, U373	ROS, ICD	Nanomolar IC_{50}, 2-photon PDT efficacy
5	[36]	Ru–Pt conjugate	Curcumin	HeLa, A2780	Chemo-PDT synergy	Nanomolar cytotoxicity, drug resistance overcome
6	[37]	Ru–Re binuclear	Coumarin	A549, HT29	Mitochondrial targeting	Selective cytotoxicity
7	[38]	Ru(II) tris-bipyridine	Curcumin	A549	Ferroptosis, ICD	Strong ROS, mitochondrial damage
8	[39]	Ru(II) triazole chelator	Coumarin	HeLa, A375	Blue-light PDT	Photoejection, apoptosis
9	[40]	Ru(II) polypyridyl	Coumarin	A549, HeLa, A431	ROS, ICD	$\Phi\Delta$ ~0.67, cytoplasmic localization
10	[41]	Ru(II) π-expansive	Curcumin	A431 skin	DNA interaction	Maintain activity under hypoxia
11	[42]	Ru(II) lysosome-localized	Coumarin	HeLa spheroids	2-photon PDT	Potent lysosomal phototoxicity
12	[43]	Ir(III) cyclometalated	Coumarin	HepG2	Mitochondrial ROS	IC_{50} 0.36 μM, apoptosis
13	[44]	Ir(III) polypyridyl	Curcumin	Multiple solid tumors	ROS, ICD	IC_{50} 0.012 μM, $\Phi\Delta$ ~0.78
14	[45]	Ir(III) nanoprodrug	Curcumin	Colorectal	ROS, ICD	Enhance solubility, tumor selectivity
15	[47]	Ir(III) NIR-activated	Coumarin	Digestive cancers	ROS, ICD	Deep tissue penetration
16	[48]	Ir(III) ferroptosis inducer	Curcumin	Colorectal	Ferroptosis-like ICD	Amplified ROS, systemic immunity
17	[50]	Ir(III) ferroptosis inducer	Coumarin	Lung cancer	Ferritinophagy	ICD, immune infiltration
18	[43]	Ir(III) cyclometalated	Coumarin	HepG2	Mitochondrial ROS, ICD	IC_{50} 0.36 μM, apoptosis
19	[44]	Ir(III) polypyridyl	Curcumin	Multiple solid tumors	ROS, ICD	IC_{50} 0.012 μM, Φ_Δ ~0.78
20	[45]	Ir(III) nanoprodrug	Curcumin	Colorectal	ROS, ICD	Enhance solubility, tumor selectivity
21	[47]	Ir(III) NIR-activated	Coumarin	Digestive cancers	ROS, ICD	Deep tissue penetration
22	[48]	Ir(III) ferroptosis inducer	Curcumin	Colorectal	Ferroptosis-like ICD	Amplify ROS, systemic immunity
23	[50]	Ir(III) ferroptosis inducer	Coumarin	Lung cancer	Ferritinophagy, ICD	Immune infiltration, tumor regression
24	[53]	Ir(III) NIR-activated	Curcumin	Solid tumors	ROS, ICD	Potent PDT efficacy under hypoxia
25	[54]	Ir(III) lysosome-targeted	Coumarin	HeLa	ROS, ICD	Lysosomal disruption, apoptosis
26	[55]	Ir(III) mitochondria-targeted	Curcumin	Colorectal	ROS, ICD	Mitochondrial damage, ICD induction
27	[56]	Ir(III) ER-localized	Coumarin	Breast cancer	ER stress, ICD	Calreticulin exposure, ATP release
28	[57]	Ir(III) nanocarrier hybrid	Curcumin	Multiple solid tumors	ROS, ICD, checkpoint synergy	Durable immune protection
29	[58]	Ru(II) nanocarrier hybrid	Coumarin	Colorectal	ROS, ICD	Enhance tumor selectivity
30	[59]	Ru(II) mitochondria-targeted	Curcumin	Lung cancer	ROS, ICD	Strong phototoxicity, immune activation
31	[60]	Ru(II) lysosome-localized	Coumarin	HeLa spheroids	2-photon PDT	Potent lysosomal phototoxicity
32	[61]	Ru(II) ER-localized	Curcumin	Gastric cancer	ER stress, ICD	Caspase-3 mediated apoptosis
33	[62]	Ru(II) nanoprodrug	Coumarin	Colorectal	ROS, ICD	Improve solubility, systemic immunity
34	[63]	Ru(II) NIR-activated	Curcumin	Breast cancer	ROS, ICD	Deep tissue penetration, immune synergy
35	[64]	Ru/Ir hybrid nanocarrier	Coumarin/Curcumin	Multiple solid tumors	ROS, ICD, checkpoint synergy	Durable immune protection, hypoxia tolerance
